# ﻿The genus *Nisotra* Baly, 1864 (Coleoptera, Chrysomeliae, Galerucinae, Alticini) in Taiwan, with redescriptions of four Asian species and notes on the immature stages of *N.gemella* (Erichson, 1834)

**DOI:** 10.3897/zookeys.1205.121928

**Published:** 2024-07-01

**Authors:** Chi-Feng Lee, Ming-Yao Chiang, Michael F. Geiser, Kuo-Hung Chuang

**Affiliations:** 1 Applied Zoology Division, Taiwan Agricultural Research Institute, Taichung 413, Taiwan Taiwan Agricultural Research Institute Taichung Taiwan; 2 Department of Life Sciences, Natural History Museum, London, UK Natural History Museum London United Kingdom; 3 Crop Environment Section, Taoyuan District Agricultural Research and Extension Station,Taoyuan, Taiwan Crop Environment Section, Taoyuan District Agricultural Research and Extension Station Taoyuan Taiwan

**Keywords:** *Hibiscusrosa-sinensis*, *Hibiscustaiwanensis*, *Hibiscustiliaceus*, Host plants, Lamiaceae, Malvaceae, *Mesonachinensis*, *Urenalobata*

## Abstract

*Nisotrachrysomeloides* Jacoby, 1885, *N.dohertyi* (Maulik, 1926), *N.gemella* (Erichson, 1834), and *Nisotranigripes* Jacoby, 1894 are redescribed with illustrations of aedeagi, antennae, gonocoxae, abdominal ventrite VIII, and spermathecae. *Nisotranigripes* is recorded for the first time from Taiwan. The immature stages and life history of *N.gemella* were studied in the laboratory using a novel rearing design. Four synonyms previously proposed are confirmed: *Sphaerodermajavana* de Motschulsky, 1866, *S.orbiculata* de Motschulsky, 1866, *Nisotrabowringi* Baly, 1876, and *Podagricahibisci* Bryant, 1941 with *N.gemella* (Erichson, 1834). Lectotypes are designated for *Halticagemella* Erichson, 1834, *N.chrysomeloides* Jacoby, 1885, *N.bowringi* Baly, 1876, and *Podagricahibisci* Bryant, 1941.

## ﻿Introduction

*Nisotra* Baly, 1864 is a widespread genus of flea beetles occurring in Oriental, Palaearctic, Australian, Madagascar, and Afrotropical regions which contains approximately 90 species (Nadein 2013). Only eight species have been recorded from the Palearctic region (Döberl 2010), of which *N.gemella* (Erichson, 1834) is the only species found in Taiwan (Kimoto and Takizawa 1997). *Nisotragemella* is one of the most widespread species of the genus, occurring west to India, east to Taiwan and the Philippines, north to China, and south to Indonesia (Sumatra and Java) (Kimoto 2000, 2001). This species was firstly recorded in Taiwan by Chen (1934a) and a number of subsequent records were reported (Kimoto 1966, 1970, 1986, 1987, 1989, 1991; Takizawa et al. 1995). Several different host plant species were recorded to *N.gemella*, including *Hibiscusrosa-sinensis* L. (Malvaceae) by Bryant (1941), Boehmerianiveavar.nivea (L.) Gaudich. (Urticaceae) by Takizawa (1979) and Yu et al. (1996), *Urenalobata* L. (Malvaceae) by Kimoto (2003), and *Gonostegiahirta* (Blume) Miq. (Urticaceae) by Takizawa (1979).

Beginning during 2005, Taiwanese populations of the genus have been collected and observed extensively by members of the Taiwan Chrysomelid Research Team (TCRT). The TCRT is composed of ten amateurs interested in producing a complete inventory of chrysomelid species in Taiwan. Adults collected from Kinmen Island (金門) were feeding on leaves of *Hibiscusrosa-sinensis*, but populations in Taiwan were found feeding on those of *H.tiliaceus* L. and *H.taiwanensis* S.Y. Hu (current study). In addition, we observed adults feeding on leaves of *Mesonachinensis* Benth. (Lamiaceae). Most of leaves have not been recorded as host plants of *N.gemella*. A taxonomic revision of Taiwanese populations was therefore deemed necessary.

**Figure 1.**
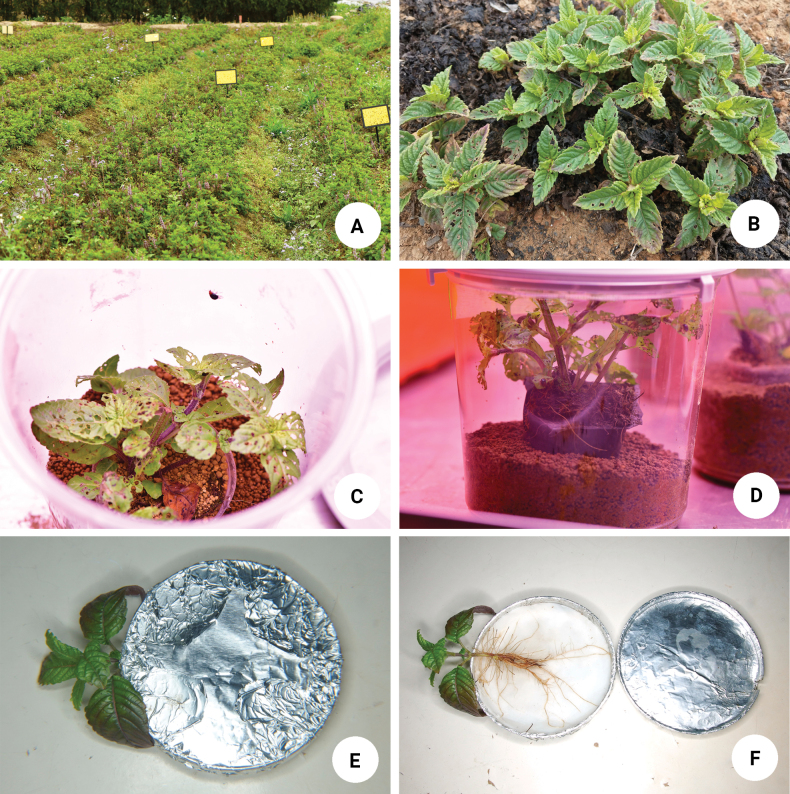
Experiments on immature stages of *Nisotragemella* (Erichson) **A** plantation of mesona (*Mesonachinensis*, 仙草), with yellow sticky insect traps for monitoring populations of *N.gemella***B** feeding marks made by adults of *N.gemella***C** young sprouts of mesona placed in plastic cups (Pint-sized BugDorm) as well as adults of *N.gemella*, in dorsolateral view **D** ditto, in lateral view **E** a special design for observing immature stages of *N.gemella* with plastic petri dishes closed **F** ditto, but plastic petri dishes opened.

*Mesonachinensis*, referred to as “mesona” (仙草), is an import crop (Fig. 1A, B) that is made into “grass jelly” (仙草凍), a dessert consumed in Taiwan, Hong Kong, Macau, Vietnam, and Southeast Asia. Grass jelly is made by boiling leaves of mesona for hours and cooling the liquid. In addition, mesona can be made into different drinks and desserts, such as hot grass jelly (燒仙草) and mesona tea (仙草茶). Recently the senior author found adults of *Nisotra* feeding on mesona and causing serious damage (Fig. 1B). Since the plants are small and easily manipulated in the laboratory, we set up some modified equipment for observing immature stages of the beetles as the immature stages of this genus are poorly known. Only *N.basselae* (Bryant, 1941), an agricultural pest of Slippery cabbage, *Abelmoschusmanihot* Medicus in the Solomon Islands, has its immature stages briefly described as part of an unpublished dissertation (Vaqalo 2014).

Identification of species of *Nisotra* is problematic due to their similar morphologies. Scherer (1969) was the first to use male aedeagi for identifying species from the Indian Subcontinent. Zhang and Yang (2007) recorded five species of *Nisotra* from China based on examination of the aedeagi and provided a key to *Nisotra* species based on external morphology. In contrast, only one species, *N.gemella*, was identified from China by Gressitt and Kimoto (1963), and Indochina by Kimoto (2000). Medvedev (2009) identified *N.chrysomeloides* and *N.gemella* based only on the male aedeagus.

For clarifying species diversity in Taiwan and species identities in Taiwan, China, and Indochina, species identities of *Nisotra* collected from Indochina and Taiwan were re-evaluated based on specimens from numerous institutions, including the following with large collections of the genus: the Natural History Museum, London, UK (**NHMUK**), the largest collection of leaf beetles globally; the Senckenberg Deutsches Enomologisches Institut, Müncheberg, Germany (**SDEI**), where specimens collected from Taiwan during the 1910s by Sauter and identified by Chen (1933) are housed; and the Kitakyushu Museum of Natural History and Human History, Kitakyushu, Japan (**KMNH**), where specimens collected from Taiwan during 1960s to 1980s identified by Kimoto are housed. Four species recorded from China, *N.gemella*, *N.chrysomeloides*, *N.dohertyi*, and *N.nigripes* are redescribed. In the former studies only aedeagi and spermathecae were studied (Zhang and Yang 2007). During this study antennae, abdominal ventrites VIII in females, and gonocoxae were also examined to evaluate their diagnostic values.

## ﻿Materials and methods

To obtain eggs of *N.gemella*, young sprouts of mesona were placed in plastic cups (Pint-sized BugDorm, vol. 720 ml) covered by net screen. Eight pairs of *Nisotra* adults were collected from Hsinpu (新埔) in Hsinchu county and placed with the sprouts (Fig. 1C, D) in one plastic cup.

For laboratory rearing of larvae, young sprouts were dug up and roots were washed with water to remove soil. Roots were then put into 9-cm diameter plastic petri dishes covered with tin foil and with blotter paper lining the bottoms (Fig. 1E, F). Eggs were put into the petri dishes and kept in darkness and constant temperature (25 ± 1 °C). Larvae were transferred to the plastic petri dishes with roots as mentioned above and observed daily.

For taxonomic study, abdomens of adults were separated from the forebodies and boiled in 10% KOH solution, followed by washing in distilled water to prepare genitalia for illustrations. The genitalia were then dissected from the abdomens, mounted on slides in glycerine, and studied and drawn using a Leica M165 stereomicroscope. For detailed examinations, a Nikon ECLIPSE 50i microscope was used.

At least three males and three females from each species were examined to delimit variability of diagnostic characters. For species collected from more than one locality or with color variations, at least one pair of each sex from each locality and color morph was examined. Length was measured from the anterior margin of the eye to the elytral apex, and width at the greatest width of the elytra. Nomenclature for morphological structures of adults follows Duckett and Daza (2004). Names of plant species follows Taiwan Encyclopedia of Life (2024), TaiEOL.

Terminology of tubercles on larvae follows Kimoto (1962) and Takizawa (1972). Tubercles are defined as sclerotized plates surrounding the bases of primary setae on the body surface. Body surface is divided into five regions: dorsal, dorso-lateral, epipleural, pleural, and sternal regions. The dorsal region has a group of tubercles named group D, which is divided into three tubercles: Da, Dpi, and Dpe, or four tubercles: Dai, Dae, Dpi, and Dpe, where subscripts ‘a’, ‘p’, ‘i’, and ‘e’ denote ‘anterior’, ‘posterior’, ‘interior’, and ‘exterior’, respectively. The dorso-lateral region has tubercle **DL**, which is sometimes divided into two separate tubercles. Epipleural regions have tubercles **EP**, which are also sometimes divided. Pleural regions have a tubercle **P**. The sternal region has three tubercles: parasternal (**PS**), sternellar (**SS**), and eusternal (**ES**) tubercles. Spiracles (**sp**) are present on meso- and metathoraces, and abdominal segments I–VIII.

Specimens studied herein are deposited at the following institutes and collections:

**KMNH** Kitakyushu Museum of Natural History and Human History, Kitakyushu, Japan [Yûsuke Minoshima]

**MCSN**Museo Civico di Storia Naturale “Giacomo Doria”, Genova, Italy [Roberto Poggi]

**MCZC**Museum of Comparative Zoology, Harvard University, Massachusetts, USA [Philip D. Perkins]

**MNHUB**Museum für Naturkunde, Leibniz-Institut für Evolutions- und Biodiversitätsforschung an der Humboldt-Universität zu Berlin, Berlin, Germany [Bernd Jäger]

**NHMUK** The Natural History Museum, London, UK [Michael F. Geiser]

**NMNS**National Museum of Natural Science, Taichung, Taiwan [Bao-Cheng Lai]

**SDEI** Senckenberg Deutsches Enomologisches Institut, Müncheberg, Germany [Mandy Shröter]

**SEHU**Laboratory for Systematic Entomology, Hokkaido University, Sapporo, Japan [Haruo Takizawa]

**TARI** Applied Zoology Division, Taiwan Agricultural Research Insitute, Taichung, Taiwan [Chi-Feng Lee]

**ZIN**Zoological Institute, Russian Academy of Science, St. Petersburg, Russia [Alexey G. Mosekyo]

Exact label data are cited for all type specimens of described species; a double slash (//) divides the data on different labels and a single slash (/) divides the data in different rows. Other comments and remarks are in square brackets: [p] – preceding data are printed, [h] – preceding data are handwritten, [w] – white label, [y] – yellow label, [g] – green label, [b] – blue label, and [r] – red label. Traditional Chinese fonts are added to the names of localities.

## ﻿Taxonomy

### ﻿ Nisotra chrysomeloides

Taxon classificationAnimaliaColeopteraChrysomelidae

Jacoby, 1885

42A3C979-E19F-54FE-8E86-3B3D785B8C58

**Figure 2.**
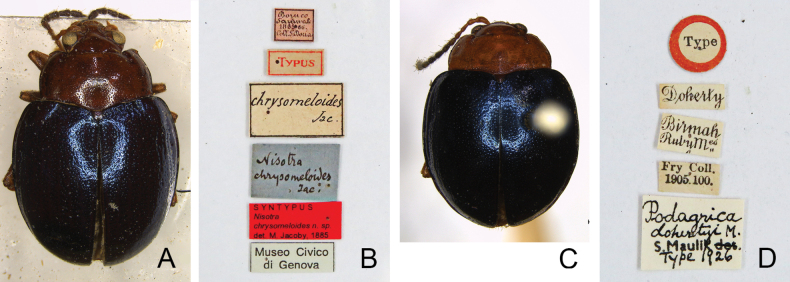
Type specimens and labels **A***Nisotrachrysomeloides* Jacoby, 1885, lectotype **B** labels pinned with lectotype **C***Podagricadohertyi* Maulik, 1926, holotype **D** labels pinned with holotype.

**Figure 3.**
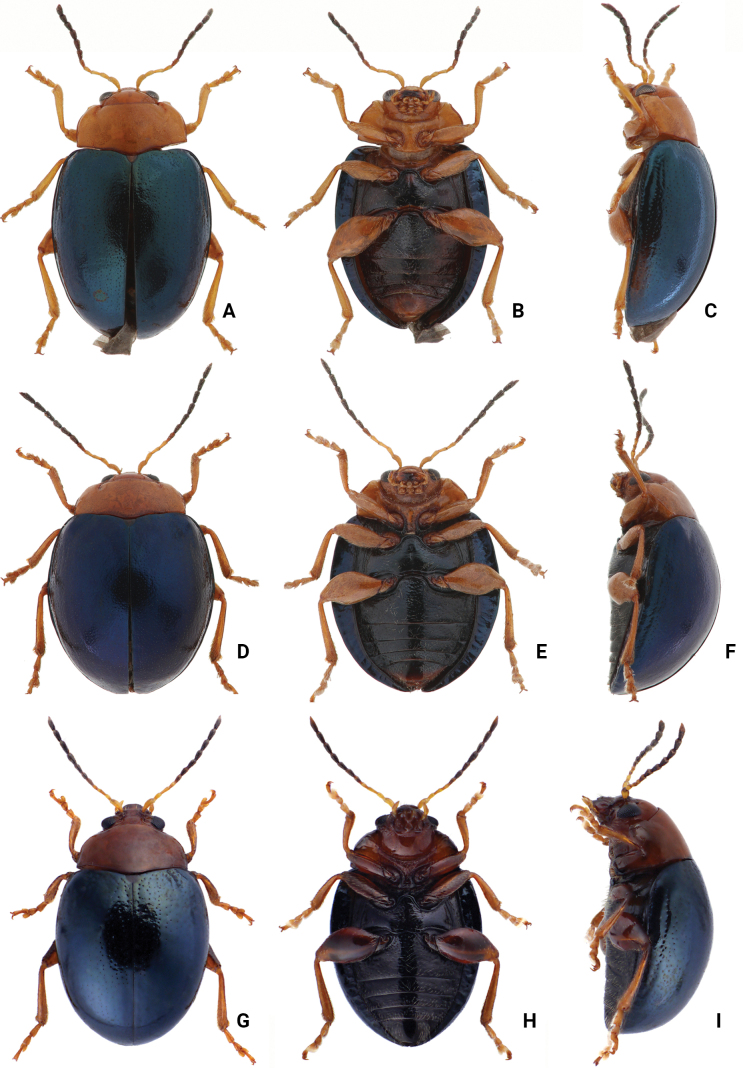
Habitus of *Nisotra* species **A***N.chrysomeloides* Jacoby, female, dorsal view **B** ditto, ventral view **C** ditto, lateral view **D***N.dohertyi* (Maulik), female, dorsal view **E** ditto, ventral view **F** ditto, lateral view **G***N.gemella* (Erichson), female, dorsal view **H** ditto, ventral view **I** ditto, lateral view.

**Figure 4.**
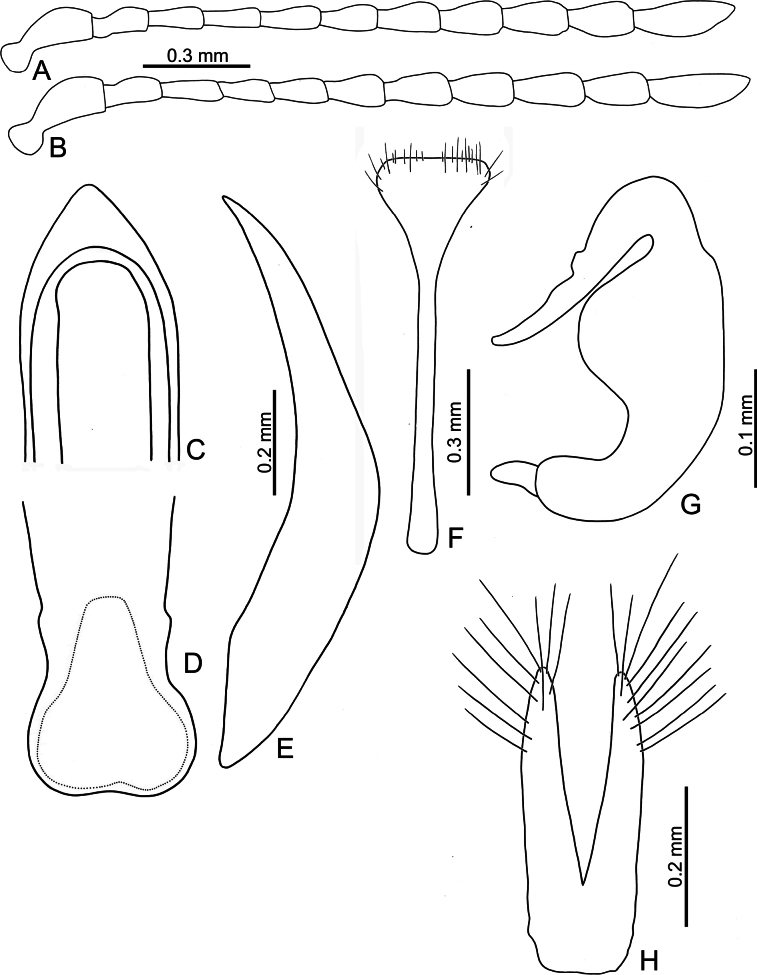
*Nisotrachrysomeloides* Jacoby, adult **A** antenna, male **B** antenna, female **C** apex of aedeagus, front view **D** base of aedeagus, dorsal view **E** aedeagus, lateral view **F** abdominal ventrite VIII, female **G** spermatheca **H** gonocoxae.

Figs 2A, B , 3A–C , 4

Nisotra chrysomeloides Jacoby, 1885: 36 (Malaysia: Sarawak); Weise 1922: 126 (Indonesia: Java; redescription); Scherer 1969: 150 (India, Myanmar, Vietnam, China; illustration of male aedeagus); Wang 1992: 684 (China: Sichuan, Yunnan); Medvedev 1992: 32 (Nepal); Medvedev 2000: 21 (Nepal).
Podagrica dohertyi : Maulik, 1926 (one paratype).
Nisotra orbiculata sensu Gressitt and Kimoto 1966: 793 (China: Hainan, Liamui).
Nisotra gemella sensu Medvedev 1993: 17 (Philippines: Palawan); Kimoto 2000: 233 (Laos: Umg. Vientiane).

#### Types.

*Nisotrachrysomeloides*. ***Lectotype*** ♂ (MCSN, here designated for clarifying its taxonomic status) (Fig. 2A, B), labeled: “Borneo / Sarawak / 1865 66. / Coll. J. Doria [p, w] // Typus [p, w, red letters, with red border] // chrysomeloides / Jac. [p, w] // Nisotra / chrysomeloides / Jac. [h, b] // SYNTYPUS / *Nisotra* / *chrysomeloides* n. sp. / det. M. Jacoby, 1885 [p, r] // Museo Civico / di Genova [p, b]”. Paralectotypes. 3♂, 4♀ (MCSN): “Borneo / Sarawak / 1865 66. / Coll. J. Doria [p, w] // SYNTYPUS / *Nisotra* / *chrysomeloides* n. sp. / det. M. Jacoby, 1885 [p, r] // Museo Civico / di Genova [p, b]”; 2♀ (MCSN): “Sarawak / Doria 65 [p, w] // SYNTYPUS / *Nisotra* / *chrysomeloides* n. sp. / det. M. Jacoby, 1885 [p, r] // Museo Civico / di Genova [p, b]”; 1♀ (NHMUK): “Borneo / Sarawak / 1865 66. / Coll. J. Doria [p, w] // Jacoby Coll. / 1909-28a [p, w] // chrysomeloides Jac [h, b] // SYN- / TYPE [p, w, circle label with blue border]”; 1 (sex undetermined, MCZC): “Borneo / Sarawak / 1865 66. / Coll. J. Doria [p, w] // 1st Jacoby / Coll. [p, w] // chrysomeloides / Jac. [h, b] // Type [p] / 18561 [h, r]”.

*Podagricadohertyi*. 1♀ (NHMUK): “Co- / type [p, w, circle label with yellow border] // Doherty [p, w] // Birmah / RubyM^es^ [p, w] // Fry Coll. / 1905. 100. [p, w] // *Podagrica* / *dohertyi* M. [h] / S. Mulik det. [p] / type 1926 [h, w]”.

#### Additional material examined.

**Cambodia.** 1♂ (NHMUK), Phnom Bokor, 24–31.X.2007, leg. S. Murzin; 1♀ (NHMUK), Kirirom, 15–17.X.2007, leg. S. Murzin; **China.** Hainan: 2♂, 2♀ (NHMUK), leg. J. Whitehead, 1899.315; 1♀ (KMNH), Liamui, 2.VIII.1933, leg. J. L. Gressitt; Hong Kong: 2♂ (KMNH), Lam Tsuen Valley nr Pak Ngau Shek, 30.V.1965, leg. Y. Miyatake; Yunnan: 1♂ (TARI), Banggunjianshan (邦棍尖山), 20.V.2016, leg. Y.-T. Wang; 2♂, 1♀ (TARI), Bakaliangzi (坝卡梁子), 26.III.2018, leg. Y.-T. Wang; 1♂, 3♀ (TARI), Bakaxiaozhai (巴卡小寨), 2.VIII.2017, leg. Y.-T. Wang; 1♂ (TARI), same but with “18.IX.2017”; 2♀ (TARI), Bangdashan (邦達山), 16.IX.2015, leg. Y.-T. Wang; 2♂, 1♀ (TARI), Bulangshan (布朗山), 28.IX.2017, leg. Y.-T. Wang; 2♀ (TARI), Chinoshan (基諾山), 30.V.2018, leg. Y.-T. Wang; 1♀ (TARI), Luteshan (綠德山), 9.IX.2017, leg. Y.-T. Wang; 2♀ (TARI), Manfen (曼粉), 12.V.2016, leg. Y.-T. Wang; 1♂ (TARI), same but with “20.IX.2017”; 1♂ (TARI), Menglun (勐侖), 2.VIII.2017, leg. Y.-T. Wang; 1♀ (TARI), Mohan (磨憨), 14.V.2016, leg. Y.-T. Wang; 2♂, 6♀ (TARI), Ruili (瑞麗), 6.IX.2014, leg. Y.-T. Wang; **India.** 2♀ (MCSN), Mungphu, 1890, leg. D. Atkinson; Sikkim: 1♀ (KMNH); Uttarakhand: 1♂ (SEHU), Mohand Forest nr Dehra Dun, 7–9.XI.1978; **Indonesia.** Java: 1♀ (SEHU), Bremi, Mt. Argopuro, 31.V.2005, leg. H. Takizawa; 1♂ (MCSN), Buitenzorg (= Bogor), 1878, leg. D. Lansberge; 1♂, 2♀ (MCSN), Sinagar, IV.1876, leg. Beccari; 6♂, 12♀ (MCSN), Tcibodas, X.1874, leg. O Beccari; Sumatra: 2♂, 4♀ (SDEI), coll. Kraatz; 1♂, 1♀ (MCSN), Balighe, X.1890–III.1891, leg. E. Modigliani; 1♂, 1♀ (SEHU), Jambi, Lapak Aur, 6.II.2006, leg. H. Takizawa; 1♀ (MCSN), Mentawei Island, Si Oban, IV.-VIII.1894, leg. Modigliani; 1♀ (MCSN), Padang, 1890, leg. E. Modigliani; 2♂, 1♀ (MCSN), Pangherang -Pisang, X.1890–III.1891, leg. E. Modigliani; 1♂, 1♀ (SEHU), Gunnung Sibayak (Tanah Karo), 11.II.1984, leg. H. Urban, K. Urban, I. Worm, J. Wiesner; 2♂, 2♀ (MCSN), Soekaranda, leg. Dohrn; **Laos.** 1♂, 1♀ (NHMUK), Haut Mekong, Muong Sing, 18.IV.1918, leg. R. V. de Salvaza; 1♀ (KMNH), Umg. Vientiane, III. –VI.1963; Champasak: 2♂, 2♀ (NHMUK), Bolaven Paleau, Rout (No. 23), Pakse-Paksong, Ban Itou env (km 35), 10–18.IV.1999, leg. E. Jendek & O. Šauša; Hua Pan: 5♂, 19♀ (NHMUK), Ban Saleui, Phou Pan Mt., 3–30.VI.2014, leg. C. Holzschuh; 3♂, 5♀ (NHMUK), same but with “27.IV. –1.VI.2011”; Louang Namtha: 1♀ (NHMUK), 20 km NW Louang Namtha, 24–30.V.1997, leg. Jendek; Phongsaly: 9♂, 4♀ (NHMUK), Phongsaly, Phu Fa, 26.VII.2006, leg. M. Geiser; Vientiane: 1♀ (NHMUK), Vang Vieng, 20.VII.2006, leg. A. Strauch; **Malaysia.** Pahang: 3♂, 2♀ (SEHU), Cameron Highland, Gn. Brinchiang, 30.VIII.2016, leg. H. Takizawa; Sabah: 1♂, 1♀ (SEHU), Kota Kinabalu, Kg. Kiapad, Inanam, 26.VI.2010, leg. H. Takizawa; 1♂, 2♀ (SEHU), Ranau, Kundasan, 30.VI.2007, leg. H. Takizawa; Sarawak: 1♀ (SEHU), Kelambit Highland, Bario, 13.VI.2018, leg. A. Abe; **Myanmar.** 3♂, 2♀ (2♂: SDEI; 1♂, 1♀: MCSN; 1♀: NHMUK), Carin Cheba, V.–VII.-(18)88, leg. L. Fea; 1♂ (NHMUK), Karen Mts., leg. Doherty, Fry Coll., 1905.100; 1♂ (NHMUK), Momeit, leg. Doherty, Fry Coll., 1905.100; 1♀ (NHMUK), Pegu, Atkinson Coll., 1892-3; 1♀ (NHMUK), Toungoo, coll. Andrewes, 1922-221; Kachin State: 2♀ (NHMUK), Nam Tamai, 2.VIII.1938, leg. R. Kaulback; **Nepal.** Bagman Zong: 1♀ (MCSN), Kathmandu valley, Lalitpur Distr., Godawari-Phulchoki, 1–7.VI.1996, leg. P. Čechovský; **Philippines.** Palawan: 1♀ (MCSN), Singapan Basin, Tau’t reservation, 11.XII.1990–5.I.1991, coll. Medvedev; **Thailand.** Nakhon Ratchasima: 1♀ (NHMUK), 15.VI.1962, coll. C. I. E.; Nan: 12♂, 11♀ (NHMUK), Doi Phuka N. P., V.2000, leg. local collector; Tak: 1♀ (NHMUK), Mae Chan / Mae Klong confluence, Thung Yai Wildlife Sanctuary, 27.IV.–6.V.1988, leg. M. J. D. Brendell; **Vietnam.** Gia Lai-Kon Tum: 1♀ (MCSN), So Lang, 50 km N Ankhe Ha-Nung, 9.XI.1979, coll. Medvedev; Lao Cai: 1♂ (NHMUK), Cha Pa (= Sa Pa), 13–20.IV.1962, leg. A. Warchalowski; Vinh Phuc: 6♂, 3♀ (NHMUK), Tam Dao, 24–26.IV.1962, leg. A. Warchalowski; 1♀ (NHMUK), same locality, 13.X.1966, leg. A. Jadwiszczak; 2♂ (NHMUK), same locality, 17.X.1966, leg. J. Kania.

#### Redescription.

**Adults.** Length 4.0–4.7 mm, width 2.6–3.2 mm (*n* = 202). General color orange or reddish brown (Fig. 3A–C); elytra, meso- and metathoracic, and abdominal ventrites metallic purple; four basal antennomeres I–IV yellowish brown, V dark brown, VI–XI black. Antennae (Fig. 4A) filiform in males, ratios of lengths of antennomeres I to XI 1.0: 0.6: 0.6: 0.6: 0.6: 0.6: 0.6: 0.6: 0.7: 0.7: 1.0; ratios of length to width from antennomeres I to XI 2.9: 2.3: 2.5: 2.8: 2.6: 2.2: 2.2: 1.8: 2.0: 1.9: 3.0; similar in females, ratios of lengths of antennomeres I to XI (Fig. 4B) 1.0: 0.5: 0.6: 0.5: 0.5: 0.5: 0.6: 0.6: 0.6: 0.6: 1.0; ratios of length to width from antennomeres I to XI 2.6: 2.2: 2.8: 2.3: 2.3: 1.8: 2.0: 1.8: 1.9: 1.9: 3.0. Pronotum 1.9–2.0 × wider than long; disc shining, with sparse, fine punctures, slightly convex; longitudinal groove on each side of apical margin deep, with several coarse punctures along groove; longitudinal groove along basal margin short and shallow; lateral margins rounded; apical margins slightly concave; basal margin medially convex. Elytra 1.2 × longer than wide; disc with coarse punctures arranged into paired longitudinal lines, with fine punctures between coarse punctures; lateral margins rounded, narrowed behind middle. Aedeagus (Fig. 4C–E) wide, ~ 3.8 × longer than wide; parallel sided, moderately narrowed at apical 1/10, apex pointed; moderately curved in lateral view; tectum sclerotized. Endophallic spiculae reduced. Gonocoxae (Fig. 4H) longitudinal and basally connected; apex of each gonocoxa widely rounded, curved dorsally, with nine or ten long setae. Ventrite VIII (Fig. 4F) well sclerotized, short setae arranged into transverse line along sides of apical margin, long setae arranged in transverse line inside apical margin, spiculum extremely long. Spermathecal receptaculum (Fig. 4G) strongly swollen; pump long and curved, with long apical process; sclerotized spermathecal duct moderately long after base of spermathecal gland.

#### Diagnosis.

**Figure 5.**
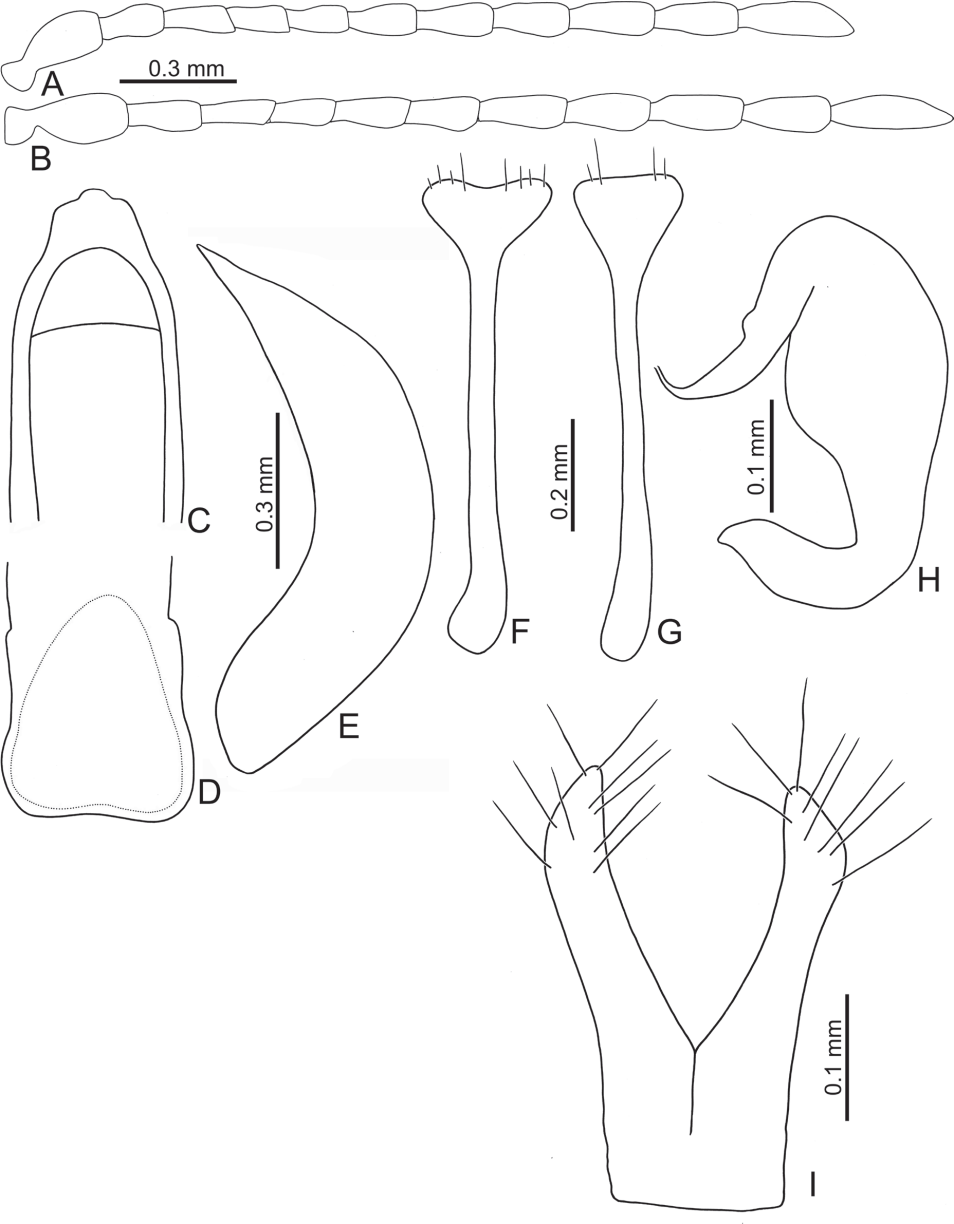
*Nisotradohertyi* (Maulik), adult **A** antenna, male **B** antenna, female **C** apex of aedeagus, front view **D** base of aedeagus, dorsal view **E** aedeagus, lateral view **F** abdominal ventrite VIII, female, from China (Yunnan) **G** abdominal ventrite VIII, female, from China (Laos) **H** spermatheca **I** gonocoxae.

**Figure 6.**
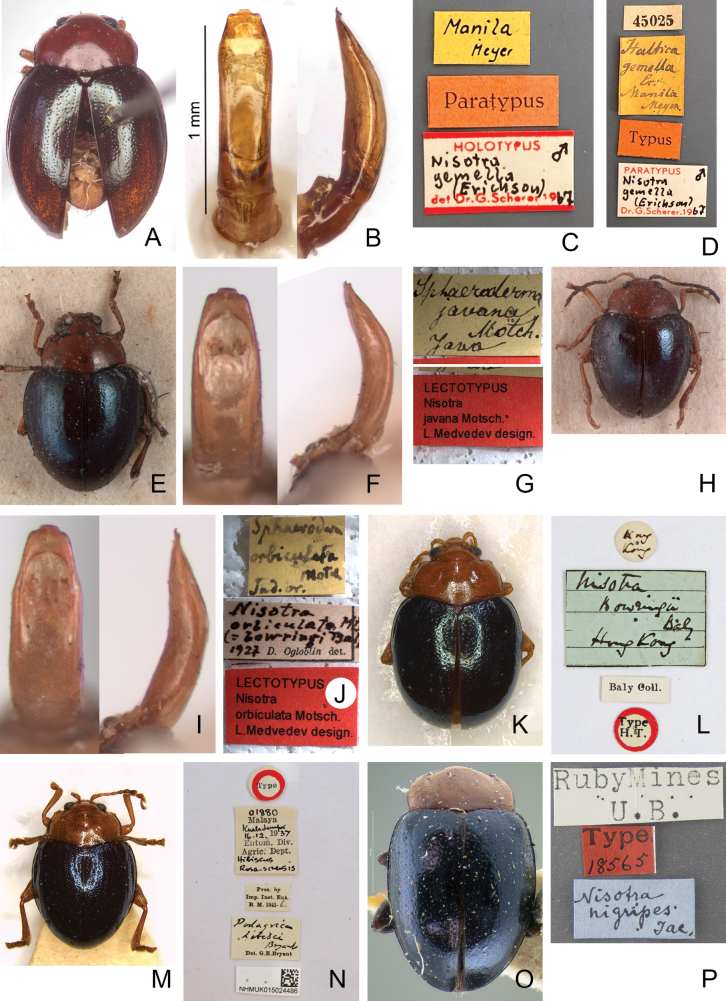
Type specimens and labels **A***Halticagemella* Erichson, 1834, lectotype **B** aedeagus dissected from lectotype, left: dorsal view; right: lateral view **C** labels pinned with lectotype **D** labels pinned with a paralectotype **E***Sphaerodermajavana* Motschulsky, 1866, lectotype **F** aedeagus dissected from lectotype, left: dorsal view; right: lateral view **G** labels pinned with lectotype **H***Sphaerodermaorbiculata* Motschulsky, 1866, lectotype **I** aedeagus dissected from lectotype, left: dorsal view; right: lateral view **J** labels pinned with lectotype **K***Nisotrabowringi* Baly, 1876, lectotype **L** labels pinned with lectotype **M***Podagricahibisci* Bryant, 1941 **N** labels pinned with lectotype **O***Nisotranigripes* Jacoby, 1894, holotype **P** labels pinned with holotype.

**Figure 7.**
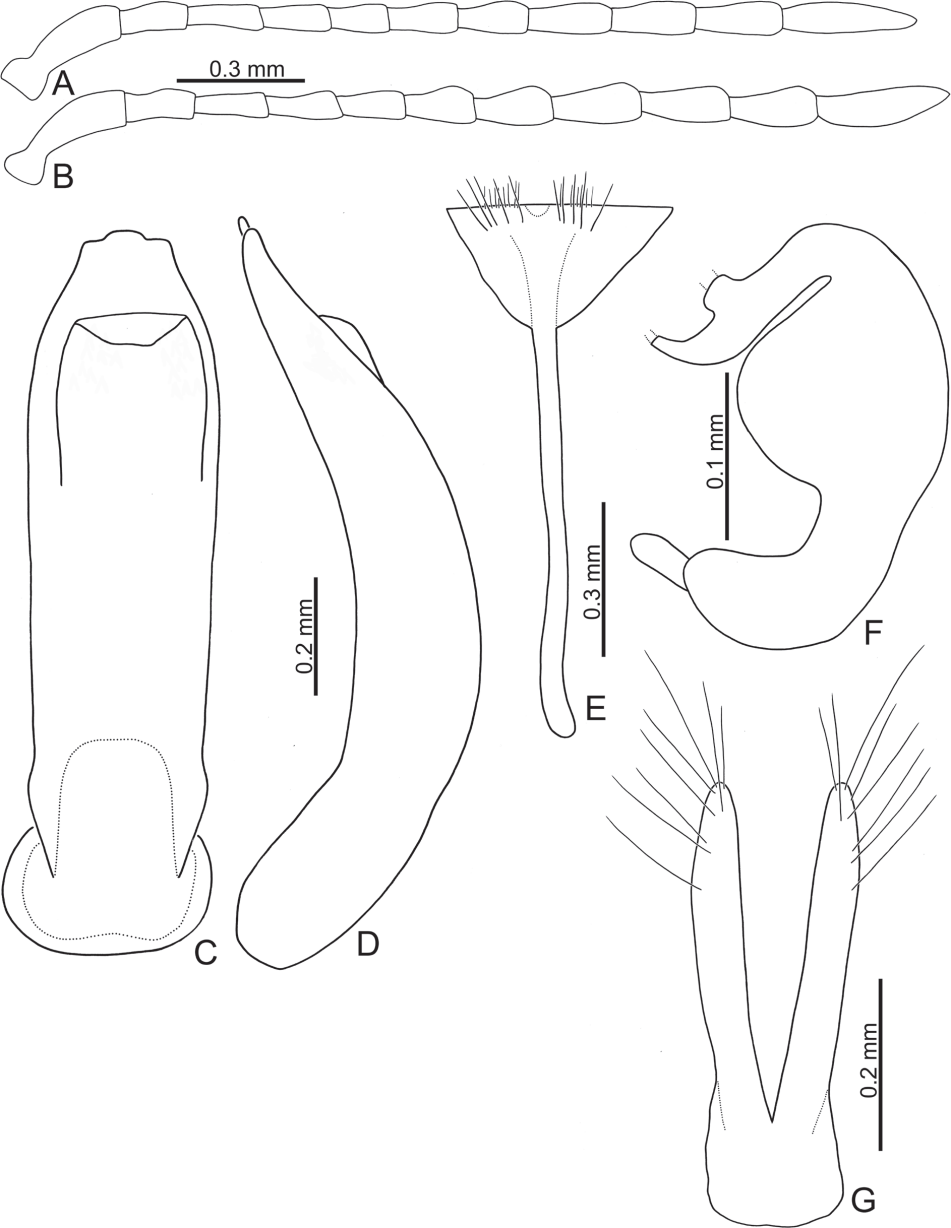
*Nisotragemella* (Erichson), adult **A** antenna, male **B** antenna, female **C** aedeagus, dorsal view **D** aedeagus, lateral view **E** abdominal ventrite VIII, female **F** spermatheca **G** gonocoxae.

Adults of *Nisotrachrysomeloides* are similar to those of *N.gemella*, with similar body shapes and color patterns, but *N.chrysomeloides* can be distinguished from *N.gemella* by the distinct longitudinal grooves at the sides of the pronotal base with punctures along the grooves (longitudinal grooves almost reduced in *N.gemella*), and less convex pronotum (more convex pronotum in *N.gemella*).The acute apex of the aedeagus in *N.chrysomeloides* (Fig. 4C) differs from the truncate apex and small process in *N.dohertyi* (Fig. 5C) and *N.gemella* (Fig. 7C), and widely rounded apex and small process at middle in *N.nigripes* (Fig. 14C). The moderately curved aedeagus in lateral view differs from the slightly curved aedeagus in *N.nigripes* (Fig. 14D) and strongly curved aedeagus in *N.dohertyi* (Fig. 5E). The sclerotized tectum (Fig. 4C) differs from the membranous tectum in others (Figs 5C, 6C, 14C). In females of *N.chrysomeloides*, the dorsally directed apices of the gonocoxae (Fig. 4H) differ from the straight gonocoxae in *N.gemella* (Fig. 7G), the laterally directed apices in *N.nigripes* (Fig. 14G), and inwardly directed apices in *N.dohertyi* (Fig. 5I). Abdominal ventrite VIII, with one transverse line of long setae inside the apical margin and dense short setae along the apical margin (Fig. 4F), differs from ventrite VIII with several pairs of long setae along the apical margin in *N.dohertyi* (Fig. 5F, G).

#### Distribution.

Cambodia, China, India, Indonesia (Java, Sumatra), Laos, Malaysia, Myanmar, Nepal, Philippines (Palawan), Thailand, Vietnam.

### ﻿ Nisotra dohertyi

Taxon classificationAnimaliaColeopteraChrysomelidae

(Maulik)

BA87CB76-C17F-57C1-A0AF-12B2F5895308

Figs 2C, D , 3D–F , 5

Podagrica dohertyi Maulik, 1926: 280 (Myanmar).
Nisotra dohertyi : Scherer 1969: 148; Medvedev 1992: 32 (Nepal); Zhang and Yang 2007: 842 (China: Yunnan).

#### Types.

***Holotype*** ♂ (NHMUK, by original designation) (Fig. 2C, D), labeled: “Type [p, w, circle label with red border] // Doherty [p, w] // Birmah / RubyM^es^ [p, w] // Fry Coll. / 1905. 100. [p, w] // *Podagrica* / *dohertyi* M. [h] / S. Mulik det. [p] / type 1926 [h, w]”. ***Paratypes*.** 2♂ (NHMUK): “Co- / type [p, w, circle label with yellow border] // Doherty [p, w] // Birmah / RubyM^es^ [p, w] // Fry Coll. / 1905. 100. [p, w] // *Podagrica* / *dohertyi* M. [h] / S. Mulik det. [p] / type 1926 [h, w]”.

#### Additional material examined.

**China.** Yunnan: 2♂ (TARI), Banggunjianshan (邦棍尖山), 15.IX.2015, leg. Y.-T. Wang; 1♂ (TARI), same but with “17.IX.2015”; 1♀ (TARI), same but with “11.VI.2017”; 1♀ (TARI), same but with “22.IX.2018”; 1♂, 6♀ (TARI), Bulangshan (布朗山), 28.IX.2017, leg. Y.-T. Wang; 3♀ (TARI), Dingiazhai (丁家寨), 27.IV.2018, leg. Y.-T. Wang; 1♀ (TARI), Ruili (瑞麗), 5.IX.2014, leg. Y.-T. Wang; 1♂, 1♀ (TARI), Wuliangshan (無量山), 11.VII.2017, leg. Y.-T. Wang; **Laos.** 1♀ (NHMUK), Haut Mekong, Muong Sing, 18.IV.1918, leg. R. V. de Salvaza; 3♀ (NHMUK), Haut Mekong, Nam Long, 26.IV.1918, leg. R. V. de Salvaza; Hua Pan: 6♂, 20♀ (NHMUK), Ban Saleui, Phou Pan Mt., 3–30.VI.2014, leg. C. Holzschuh; 2♀ (NHMUK), same but with “27.IV. –1.VI.2011”; 1♂ (NHMUK), Phongsaly, Phu Fa, 26.VII.2006, leg. M. Geiser; **Myanmar.** 1♀ (NHMUK), Sadon, 28.VI. –5.VII.1939, leg. R. Malaise; **Vietnam.** Lao Cai: 4♂, 3♀ (NHMUK), Cha Pa (= Sa Pa), 13–20.IV.1962, leg. A. Warchalowski; 1♀ (NHMUK), same locality, 24.IX.2004, leg. M. Geiser.

#### Redescription.

**Adults.** Length 4.0–4.4 mm, width 2.8–3.3 mm (*n* = 59). General color yellowish brown (Fig. 3D–F); elytra, meso- and metathoracic, and abdominal ventrites metallic purple; four basal antennomeres I–IV yellowish brown, V dark brown, VI–XI black . Antennae (Fig. 5A) filiform in males, ratios of lengths of antennomeres I to XI 1.0: 0.6: 0.6: 0.5: 0.6: 0.6: 0.7: 0.7: 0.8: 0.8: 1.1; ratios of length to width from antennomeres I to XI 2.4: 2.0: 2.3: 2.0: 2.2: 2.2: 2.3: 2.3: 2.5: 2.5: 3.5; similar in females, ratios of lengths of antennomeres I to XI (Fig. 5B) 1.0: 0.6: 0.6: 0.5: 0.6: 0.6: 0.7: 0.7: 0.8: 0.7: 1.0; ratios of length to width from antennomeres I to XI 2.5: 2.4: 2.6: 2.5: 2.6: 2.1: 2.6: 2.3: 2.5: 2.4: 3.7. Pronotum 2.0–2.2 × wider than long; disc dull, with micro-reticulation, less convex; with sparse, fine punctures; longitudinal groove on each side of apical margin deep, with several coarse punctures along groove; longitudinal groove on basal margin short and shallow; lateral margins rounded; apical margins slightly concave; basal margin medially convex. Elytra 1.1 × longer than wide; disc with coarse, confused punctures, mixed with fine punctures; lateral margins rounded, narrowed behind middle. Aedeagus (Fig. 5C–E) wide, ~ 3.7 × longer than wide; parallel sided, moderately narrowed at apical 1/10; apex truncate, but with one median rounded process; extremely strongly curved in lateral view; tectum membranous. Endophallic spiculae reduced. Gonocoxae (Fig. 5U) longer than wide, and basally connected; apex of each gonocoxa widely rounded, curved inwards, with eight or nine long setae. Ventrite VIII (Fig. 5F, G) well sclerotized, 2–4 pairs of setae arranged into transverse line along sides of apical margin, spiculum extremely long. Spermathecal receptaculum (Fig. 5H) strongly swollen; pump long and curved, with long apical process; spermathecal duct sclerotized, moderately long after base of spermathecal gland.

#### Diagnosis.

Adults of *Nisotradohertyi* are characterized by the confused punctures on the elytra (punctures arranged into paired longitudinal lines in other species), dull pronotum with micro-reticulation (shining pronota without micro-reticulation in others), and ovate elytra, 1.1 × longer than wide (oblong elytra, 1.2 × longer than wide in others), although this character is similar in a few adults of *N.chrysomeloides* and *N.gemella* with more ovate bodies. In males of *N.dohertyi*, the truncate apex and small process of the aedeagus (Fig. 5C) differ from the acute apex in *N.chrysomeloides* (Fig. 4C) and widely rounded apex and small medial process in *N.nigripes* (Fig. 14C). The strongly curved aedeagus in lateral view (Fig. 5E) differs from the moderately curved aedeagus in *N.chrysomeloides* (Fig. 4E) and *N.gemella* (Fig. 7D), and slightly curved aedeagus in *N.nigripes* (Fig. 14D). The membranous tectum (Fig. 5C) differs from the sclerotized tectum in *N.chrysomeloides* (Fig. 4C). In females of *N.dohertyi*, the inwardly directed apices of the gonocoxae differ from the straight gonocoxae in *N.gemella* (Fig. 7G), dorsally directed apices in *N.chrysomeloides* (Fig. 4H), and laterally directed apices in *N.nigripes* (Fig. 14G). Abdominal ventrite VIII with several pairs of long setae along apical margin (Fig. 5F, G) differs from the presence of one transverse line of long setae inside the apical margin and dense short setae along the apical margin in others (Figs 4F, 7E, 14E).

#### Distribution.

China, Laos, Myanmar, Vietnam. Records in Nepal need further confirmation.

### ﻿ Nisotra gemella

Taxon classificationAnimaliaColeopteraChrysomelidae

(Erichson)

D0EAB86A-1C67-5A85-A205-BB7AF96D507B

Figs 3G–I , 6A–N , 7 , 8 , 9 , 10 , 11 , 12

Haltica gemella Erichson, 1834: 275 (Philippines: Luzon).
Nisotra gemella : Jacoby 1885: 34 (Indonesia: Java); Scherer 1969: 148 (illustration of male aedeagus); Kimoto and Takizawa 1973: 177 (Nepal); Scherer 1979: 138 (India, Bhutan); Gruev 1985: 40 (Nepal); Kimoto 1986: 60 (additional records in Taiwan); Kimoto 1987: 191 (additional records in Taiwan); Kimoto 1989: 263 (additional records in Taiwan); Kimoto 1991: 16 (additional records in Taiwan); Medvedev 1993: 17 (Philippines: Luzon, Palawan (misidentification!)); Kimoto 2000: 233 (Laos, Thailand); Medvedev 2000: 21 (Nepal); Kimoto 2001: 201 (catalogue); Aston 2009: 5 (China: Hong Kong).
Nisotra gemellata [sic!]: Duvivier 1885: 49 (Indonesia: Sumatra).
Sphaeroderma javana Motschulsky, 1866: 421 (Indonesia: Java). Synonymized with N.gemella by Medvedev (2006). Synonym confirmed.
Podagrica javana : von Harold 1876: 3481.
Nisotra javana : Weise 1922: 126; Ogloblin 1930: 106.
Sphaeroderma orbiculata Motschulsky, 1866: 421 (India); Harold 1876: 3548 (catalogue). Synonymized with N.gemella by Scherer (1969). Synonym confirmed.
Nisotra orbiculata : Ogloblin 1930: 106; Chen 1933: 55 (China: Guandong); Chen 1934a: 181 (Taiwan); Chen 1934b: 278 (South China, Vietnam, India); Chûjô 1935: 474 (catalogue of Taiwan fauna); Gressitt and Kimoto 1963: 793 (China: Sichuan, Jiangxi, Guanxi, Fujian, Hainan Island); Kimoto 1966: 35 (additional records in Taiwan); Scherer 1969: 148 (India); Kimoto 1970: 215 (New Taipei City: Pinglin (坪林)); Takizawa et al. 1995: 14 (additional records in Taiwan).
Nisotra bowringi Baly, 1876: 584 (China: Hong Kong); Jacoby 1889: 196 (Myanmar). Synonymized with N.orbiculata by Ogloblin (1930). Synonym confirmed.
Podagrica bowringi : Maulik 1926: 278 (India).
Podagrica hibisci Bryant, 1941: 286 (Malaysia; host plant: Hibiscusrosa-sinensis L.). Synonymized with N.gemella by Scherer (1969). Synonym confirmed.

#### Types.

*Halticagemella*. ***Lectotype*** ♂ (MNHUB, here designated to preserve stability of nomenclature), labeled (Fig. 6A–C): “Manila / Meyer [h, y] // Paratypus [p, r] // HOLOTYPUS [p, red letters] / Nisotra ♂ / gemella / (Erichson) [h, black letters] / det Dr. G. Scherer 19 [p, red letters] 67 [h, w, black letters]”. Paratypes (MNHUB): three specimens bear same labels as lectotype except “PARATYPUS [p, red letters] / Nisotra ♂ or ♀ / gemella / (Erichson) [h, black letters] / Dr. G. Scherer 19 [p, red letters] 67 [h, w, black letters]”. One specimen labeled (Fig. 6D): “45025 [p, w] // Haltica / gemella / Eri / Manila / Meyer [h, y] // Typus [p, r] // PARATYPUS [p, red letters] / Nisotra ♂/ gemella / (Erichson) [h, black letters] / Dr. G. Scherer 19 [p, red letters] 67 [h, w, black letters]”. Although labels for holotype and paratypes were written by Scherer, but it was not officially published.

*Sphaerodermajavana*. ***Lectotype*** ♂ (ZIN, designated by Medvedev 2006) (Fig. 6E–G): “Sphaeroderma / javana / Motsch. / Java [h, y] // LECTOTYPUS / Nisotra / javana Motsch. / L. Medvedev design. [p, r]”. Medvedev (2006) indicated that there are three paralectotypes female bearing same labels.

*Sphaerodermaorbiculata*. ***Lectotype*** ♂ (ZIN, designated by Medvedev 2006) (Fig. 6H–J): “Sphaeroderma / orbiculata / Motsch / Ind. or. [h, y] // Nisotra / orbiculata Mts / (= borwing Baly / 1927 [h] D. Ogloblin det. [p. w] // LECTOTYPUS / Nisotra / orbiculata Motsch. / L. Medvedev design. [p, r]”.

*Nisotrabowringi*. ***Lectotype*** ♀ (NHMUK, here designated to preserve stability of nomenclature) (Fig. 6K, L), labeled: “Hong / Kong [h, w, circle label] // Nisotra / Bowringi / Baly / Hong Kong [h, b] // Type / H. T. [p, w, circle label with red border]”. ***Paralectotype***: 1♀ (NHMUK), shared with the same pin as lectotype.

*Podagricahibisci*. ***Lectotype*** ♀ (NHMUK, here designated to preserve stability of nomenclature) (Fig. 6M, N), labeled: “01880 [h] / Malaya [p] / Kula Lumpur / 16.12. [h] 19 [p] 37 [h] / Entom. Div. [p] / Hibiscus / Rosa-sinensis [h, w] // Type [p, r, circle label with red border] // Pres. by / Imp. Inst. Ent. / B. M. 1941-61. [p, w] // Podagrica / hibisci / Bryant [h] / Det. G. E. Bryant [p, w] // NHMUK015024484 [p, w, with QR-Code]”. ***Paralectotype*.** 1♀ (NHMUK), bearing same labels as lectotype but without type and QR-Code labels.

#### Additional material examined.

**China.** Fujian: 1♀ (TARI), Chishui (赤水), 20.VI.2014, leg. Y.-T. Chung; 1♂ (KMNH), Chungan: Bohea Hills, 11.I.1940, leg. T. C. Maa; 1♀ (TARI), Zhaizhong (宅中), 24.VIII.2014, leg. Y.-T. Wang; Guandong: 1♀ (KMNH), Fei-ha to Fei-loi, 1.VII.1950, leg. J. L. Gressitt; 1♀ (NHMUK), nr. Canton (= Guanzhou, 廣州), 27.IX.1980, leg. P. M. Hammond, B.M. 1980-491; Guanxi: 1♂ (NHMUK), Huanjiang Xian, Maonan, leg. M. Häckel; Hainan: 1♀ (TARI), Nanhsitsum (南喜村), 15.XI.2018, leg. Y.-T. Wang; Hong Kong: 1♂, 1♀ (NHMUK), coll. Walker, 1893—58; Hunnan: 1♀ (NHMUK), mts. Daiongshan, Xinhua, VII.2004, leg. Jing; Yunnan: 1♂ (TARI), Bakaxiaozhai (巴卡小寨), 1.VIII.2017, leg. Y.-T. Wang; 1♂ (TARI), Chinoshan (基諾山), 31.V.2018, leg. Y.-T. Wang; 4♂ (TARI), Manfen (曼粉), 20.IX.2017, leg. Y.-T. Wang; 1♀ (TARI), Menglun (勐侖), 2.VIII.2017, leg. Y.-T. Wang; 1♀ (TARI), same but with “20.IV.2018”; 2♀ (TARI), same but with “2.V.2019”; 1♀ (TARI), Mohan (磨憨), 14.V.2016, leg. Y.-T. Wang; 1♀ (TARI), Nabang (那邦), 25.IX.2018, leg. Y.-T. Wang; 2♀ (TARI), Ruili (瑞麗), 15.IX.2014, leg. Y.-T. Wang; Zhejiang: 3♂ (NHMUK), Da-Laen-Saen, near Ning-Po, col. Walker, 1893-18; 2♂ (NHMUK), same locality, leg. J. J. Waliker, G. C. Champion Coll., B.M. 1927-409; **India.** Andaman Islands: 3♂ (NHMUK), Capt. Wimberley, Fry Coll. 1905 100; 2♂, 1♀ (NHMUK), same locality, leg. F. A. de Roepstorff, 1884-15; 1♂, 1♀ (NHMUK), Port Blair, 10.X.1989, leg. B. S. Bhumannava, feeding on *Urenalobata*; Assam: 1♀ (KMNH), Kaziranga nödl. Mikir-Hills, Brahmaputra, V.1961, leg. G. Scherer; Sikkim: 1♀ (NHMUK), Dikchu, 23.IV.1924, leg. R. W. G. Hingaton; Tamil Nadu: 5♂ (SEHU), Nilgiri, Mettupalayam, 30.II.1978; Uttarakhand: 1♀ (NHMUK), Ranikhet, Kumaon, coll. H. G. Champion, 1953-156; 1♂ (SEHU), Mohand Forest nr Dehar Dun, 7–9.XI.1978; 3♂, 1♀ (SEHU): FRI, Dehra Dun, 10–13.XI.1978; **Indonesia.** Java: 1♀ (SEHU), Bremi, Mt. Argopuro, 31.V.2005, leg. H. Takizawa; 9♂, 12♀ (MCSN), Buitenzorg (= Bogor), 1875, leg. G. B. Ferrari; 1♀ (MCSN), same but with “X.1872”; 1♀ (MCSN), same locality, 1878, leg. D. Lansberge; 1♂, 1♀ (SDEI); Sulawesi: 1♀ (SEHU), Kendari: Amoito, 1.XII.1974, leg. K. Kusigemati; 1♂ (SEHU), Kendari: Andonuhu, 3.XII.1974, K. Kusigemati; 3♂, 3♀ (MCSN), Kandari, III.(18)74, leg. O. Beccari; Sumatra: 2♂ (SEHU), Aceh, Kota Cane, 26–28.IV.1998, leg. H. Takizawa; 3♂, 4♀ (MCSN), Ayer Manicior (= Ajer Mantjoer), VIII.1878, leg. O. Beccari; 2♂, 1♀ (SDEI), Bangkei Island, 1885, leg. H. Kühn; 1♀ (MCSN), Benculen, IV.1891, leg. E. Modigliani; 1♂, 1♀ (SEHU), Bukit Gompong, Sukarami, NE 20 km from Padang, 19.VIII.1998, leg. M. Ohara; 1♂ (SEHU), Jambi, Lapak Aur, 6.II.2006, leg. H. Takizawa; 1♂ (MCSN), Padang, 1890, leg. E. Modigliani; 1♂ (MCSN), Sing Hara, X.1878, leg. O. Beccari; 1♂, 2♀ (SDEI), Tebing Tinggi, leg. Schultheiss; **Laos.** 3♂, 4♀ (NHMUK), Betw. Vientiane & Luang Prabang, end of 1919, leg. R. V. de Salvaza; Attapu: 1♂ (NHMUK), Bolaven Plateau, 15 km SE of Ban Huang Kong, Nong Lom (lake), 18–30.IV.1999, leg. E. Jendek & O. Šauša; Bolikhamsai: 1♂, 1♀ (NHMUK), Lak Sao, 18.VIII.2004, leg. M. Geiser; Borikhan: 1♂ (NHMUK), Borikhan env., 20 km N of Muang Pakxan, 18.V.2003, leg. O. Šafránek; Champasak: 1♀ (NHMUK), Bolaven Paleau, Rout (No. 23), Pakse-Paksong, Ban Itou env (km 35), 10–18.IV.1998, leg. E. Jendek & O. Šauša; Hua Pan: 1♀ (NHMUK), Ban Saleui, Phou Pan Mt., 11.IV.-15.V.2012, leg. C. Holzschuh; 1♂ (NHMUK), same but with “27.IV. –1.VI.2011”; Phongsaly: 1♂ (NHMUK), Phongsaly, Phu Fa, 26.VII.2006, leg. M. Geiser; Savannakhét: 1♂ (SEHU), 1.V.2006, leg. K. Maruyama; Vientiane: 1♀ (SEHU), Vang vieng, 28–29.V.2004, leg. T. Tsuru; 1♀ (NHMUK), same locality, 20.VII.2006, leg. M. Geiser; 2♂, 1♀ (NHMUK), same but with “21.VII.2006”; **Malaysia.** Kuala Lumpur: 1♂ (SEHU), Labu, 31.III.2007, leg. H. Takizawa; Perak: 4♂, 2♀ (MCSN), 25 km NE of Ipoh, Banjaran Titi Wangsa mts., Korbu mt., 27.I.–2.II.1999, leg. P. Čechovský; 3♂, 2♀ (MCSN), same but with “4–13.III.1998”; Sabah: 1♂, 2♀ (SDEI), Kinabalu, S. W. 11, leg. H. Rolle; **Myanmar.** 1♂, 1♀ (SDEI), Carin Cheba, V.–VII.-(18)88, leg. L. Fea; 1♂, 1♀ (NHMUK), same locality, coll. Fry, 1905.100; 1♀ (MCSN), Carin, Asciuii Cheba, I.(18)88, leg. L. Fea; 4♂, 4♀ (SDEI), Palon, Pegu, VIII.–IX.(18)87, leg. L. Fea; 1♀ (NHMUK), Momeit, leg. Doherty, Fry Coll., 1905.100; 1♀ (NHMUK), Paungde, coll. Andrewes, 1922-221; 1♂, 2♀ (NHMUK), Prome, coll. Andrewes, 1922–221; 2♂, 2♀ (SDEI), Rangoon (= Yangon), 1887, leg. Fea; 1♀ (NHMUK), same but with “Atkinson Coll., 1892–3”; 2♀ (NHMUK), same but with “coll. Fry, 1905.100”; 1♂ (NHMUK), same locality, 1933–34, leg. F. J. Meggitt; 4♂, 4♀ (4♂, 2♀: SDEI; 1♀: MCSN; 1♀: NHMUK), Teinzo, V.1886, leg. Fea; 1♂, 2♀ (NHMUK), Tharrawaddy, leg. H. Swale, 1913-117; 4♀ (NHMUK), same locality, leg. H. E. Andrewes, 1922-221; 2♂, 3♀ (NHMUK), same locality, 26.XII.1953, leg. H. G. Champion; 1♀ (NHMUK), Toungoo, 26.XII.1953, leg. H. G. Champion; Kachin State: 1♂ (MCSN), VIII.1885, leg. Fea; 1♀ (NHMUK), Bhamo, VIII.1885, leg. Fea; 2♂, 1♀ (NHMUK), Nam Tamai, 2.VIII.1938, leg. R. Kaulback; Kayin Sate: 1♀ (TARI), Than Daung Gyi, 18.V.2017, leg. Y.-T. Wang; Yangon: 2♂, 2♀ (NHMUK), Shwentha, 23.VII.1988, leg. T. T. Nwe, collected from *Urenalobata*; **Nepal.** 3♂, 2♀ (SEHU), Balaju Kathmandu Valley, 11.IX.1987, leg. H. Takizawa; **Papua New Guinea.** 1♀ (SDEI), Haveri, VII.–XI.(18)93, leg. Loia; 1♀ (SDEI), Ighibirei, VII.-VIII.(18)90, leg. Loria; 1♂ (TARI), West Highland, Begesin Missions station 43 km 237° von Madan, Kulturlandschan, 6.V.1996, leg. H. Deumer; **Philippines.** Luzon. Kalinga: 1♂ (MCSN), Tulgao, 23.VI.1988, coll. Medvedev; **Singapore.** 3♂, 1♀ (NHMUK), leg. C. J. Saunders, B. M. 1933-227; **Taiwan.** Hsinchu: 3♂, 3♀ (TARI), Hsinpu (新埔), 2.I.2010, leg. K.-H. Chuang; 18♂, 23♀ (TARI), same but with “5.X.2021”; 12♂, 14♀ (TARI), same but with “18.XII.2021”; Kinmen: 2♂, 7♀ (TARI), Kinmen Island (金門島), 18.VI.2014, leg. Y.-T. Chung; 4♂, 8♀ (TARI), same island, Jhongshanlin (中山林), 28.VI.2023, leg. C.-F. Lee; 5♀ (TARI), 太武苗圃(= Kinmen Botanical Gardens, 金門植物園), 24.IV.2002, leg. H. T. Shih; 1♀ (TARI), same but with “Taiwushan (太武山)”; Matsu: 1♀ (TARI), Beigan Island (北竿島), 14.V.2018, leg. H.-T. Fang; New Taipei City: 1♀ (KMNH), Pinglin (坪林), 23.VI.1965, leg. Y. Kurosawa; 4♂ (TARI), Watzuwei (挖仔尾), 28.VI.2008, leg. H. Lee; 1♂ (KMNH), Urai (烏來), 31.V.1976, leg. H. Makihara; Nantou: 1♀ (SDEI), Fuhosho (= Wucheng, 五城), IX.1909, leg. Sauter; Pingtung: 1♂, 1♀ (TARI), Shantimen (三地門), 1–5.III.1982, leg. K. C. Chou & C. C. Pan; 1♀ (TARI), Tahan trail (大漢林道), 10.IV.2023, leg. J.-C. Chen; Taipei: 1♀ (SDEI), Hokuto (= Peitou, 北投), III.1912, leg. Sauter; Taitung: 2♀ (SDEI), Paroe (= Tawu, 大武), IX.1912, leg. H. Sauter; Taoyuan: 1♀ (TARI), Tayuan (大園), 15.VIII.2011, leg. L.-F. Chu; **Thailand.** Chiang Mai: 1♀ (NHMUK), Fah Luan Univ. Campus, 20–22.VII.2009, leg. D. Quicke & B. et R. Butcher; 1♀ (NHMUK), Queen Sirikit Botanic Gardens, VII.2006, leg. H. Mendel & M. V. L. Barclay; 1♂ (NHMUK), Tha Ton Env., 20.IV.2003, leg. O. Šafránek; Kanchanaburi: 1♂, 1♀ (NHMUK), Thongpapoom, 11–12.VII.2009, leg. D. Quicke & B. et R. Butcher; Mae Hong Son: 1♂ (NHMUK), Mae Hong Son, 17–21.VI.1993, leg. Schneider; Nakhon Nayok: 2♂, 1♀ (SEHU), Ban Na, 27.VII.1997, leg. S. Ohmomo; Nan: 1♂, 2♀ (NHMUK), Doi Phuka N. P., V.2000, leg. local collector; Songkhla: 1♀ (NHMUK), Chon Thong, 24–27.IV.1991, leg. L. Dembický; Yala: 1♂ (NHMUK), Betong, Gunung Cang Dun vill., 25.III. –22.IV.1993, leg. J. Horák; **Vietnam.** Băc Giang: 2♂, 2♀ (TARI), Tây Yên Tů, 2.VI.2014, leg. Y.-T. Wang; Hà Nôi: 1♂, 1♀ (TARI), Huyên Mê Linh, 30.V.2014, leg. Y.-T. Wang; Hòa Binh: 3♀ (NHMUK), VIII.1918, leg. R. V. de Salvaza; 1♂ (MCSN), Ha Son-Binh, 10 km SW Hoa Binh, 17.X.1976, leg. L. Medvedev; Lao Cai: 2♂ (NHMUK), Bao Ha, 12.IV.1962, leg. A. Warchalowski; 1♂ (NHMUK), same but with “4.IV.1962”; 2♂, 5♀ (NHMUK), Cha Pa (= Sa Pa), 14.IV.1962, leg. A. Warchalowski; Nam Dinh: 1♀ (NHMUK), Van Diem, 19.III.1962, leg. A. Warchalowski; 1♂ (NHMUK), same but with “7.II.1962”; Ninh Binh: 1♂ (NHMUK), Cuc Phuong, 8.VI.1966, leg. R. Bizlawski & B. Pisarski; Quang Ninh: 1♂ (TARI), 10 km SE Tien Yen, 1–14.IV.2004, leg. H. Mühle; Vinh Phuc: 1♂, 2♀ (NHMUK), Tam Dao, 25.IV.1962, leg. A. Warchalowski; 1♂, 1♀ (NHMUK), same locality, 17.X.1966, leg. J. Kania.

#### Redescription.

**Adults.** Length 3.1–3.9 mm, width 2.0–2.5 mm (*n* = 376). General color yellowish brown (Fig. 3G–I); elytra, meso- and metathoracic, and abdominal ventrites metallic purple; four basal antennomeres I–IV yellowish brown, V dark brown, VI–XI black. Antennae (Fig. 7A) filiform in males, ratios of lengths of antennomeres I to XI 1.0: 0.5: 0.6: 0.5: 0.5: 0.5: 0.5: 0.6: 0.6: 0.7: 1.0; ratios of length to width from antennomeres I to XI 3.2: 2.4: 2.9: 2.6: 2.2: 2.2: 2.2: 2.6: 2.6: 2.6: 4.2; similar in females, ratios of lengths of antennomeres I to XI (Fig. 7B) 1.0: 0.5: 0.5: 0.5: 0.5: 0.5: 0.6: 0.6: 0.6: 0.6: 1.0; ratios of length to width from antennomeres I to XI 3.6: 2.3: 2.9: 2.5: 2.4: 1.9: 2.0: 2.2: 2.6: 2.2: 3.8. Pronotum 1.8–1.9 × wider than long; disc shining, moderately convex, with sparse, fine punctures; longitudinal groove on each side of apical margin reduced; longitudinal groove on basal margin short and shallow; lateral margins rounded; apical margins slightly concave; basal margin medially convex. Elytra 1.2 × longer than wide; disc with coarse punctures arranged into longitudinal lines, with fine punctures between coarse punctures; lateral margins rounded, narrowed behind middle. Aedeagus (Fig. 7C, D) wide, ~ 4.8 × longer than wide; parallel sided, but slightly wider at apical 1/7, and then moderately and apically narrowed; apex truncate, but with one median rounded process; strongly curved in lateral view, apex directed upward; tectum membranous, with several stout setae at apex of internal sac. Endophallic spiculae reduced. Gonocoxae (Fig. 7G) longer than wide, and basally connected; apex of each gonocoxa widely rounded, with eight long setae along apical margin and outer margin. Ventrite VIII (Fig. 7E) with apex weakly sclerotized, with one semicircular membranous area at middle of apical margin, several long setae arranged into transverse line near apical margin, and several shorter setae along apical margin, both types of setae absent medially. Spiculum extremely long. Spermathecal receptaculum (Fig. 6F) strongly swollen; pump long and curved, with long apical process; sclerotized spermathecal duct moderately long after base of spermathecal gland.

**Variations.** Adults in some populations have more ovate body shapes (elytra 1.1 × as long as wide), including those on the Andaman Islands (India).

***Remarks*.** Adults of *N.gemella* are characterized by more convex pronotum and reduced longitudinal grooves on the sides of the apical margins (less convex pronotum and long, deep longitudinal grooves on sides of apical margins in others). In males of *N.gemella*, the truncate apex of the aedeagus bears a small process (Fig. 7C) that differs from the acute apex in *N.chrysomeloides* (Fig. 4C) and the widely rounded apex and small medial process in *N.nigripes* (Fig. 14C). The moderately curved aedeagus in lateral view (Fig. 7D) differs from the strongly curved aedeagus in *N.dohertyi* (Fig. 5E) and slightly curved aedeagus in *N.nigripes* (Fig. 14D). The membranous tectum (Fig. 7C) differs from the sclerotized tectum in *N.chrysomeloides* (Fig. 4C). In females of *N.gemella*, the straight gonocoxae (Fig. 7G) differ from the dorsally directed gonocoxal apices in *N.chrysomeloides* (Fig. 4H), the inwardly directed apices in *N.dohertyi* (Fig. 5I), and laterally directed apices in *N.nigripes* (Fig. 14G). Abdominal ventrite VIII, with one transverse line of long setae inside the apical margin and dense short setae along the apical margin (Fig. 7E), differs from ventrite VIII of *N.dohertyi* (Fig. 5F, G), with several pairs of long setae along the apical margin.

**Figure 8.**
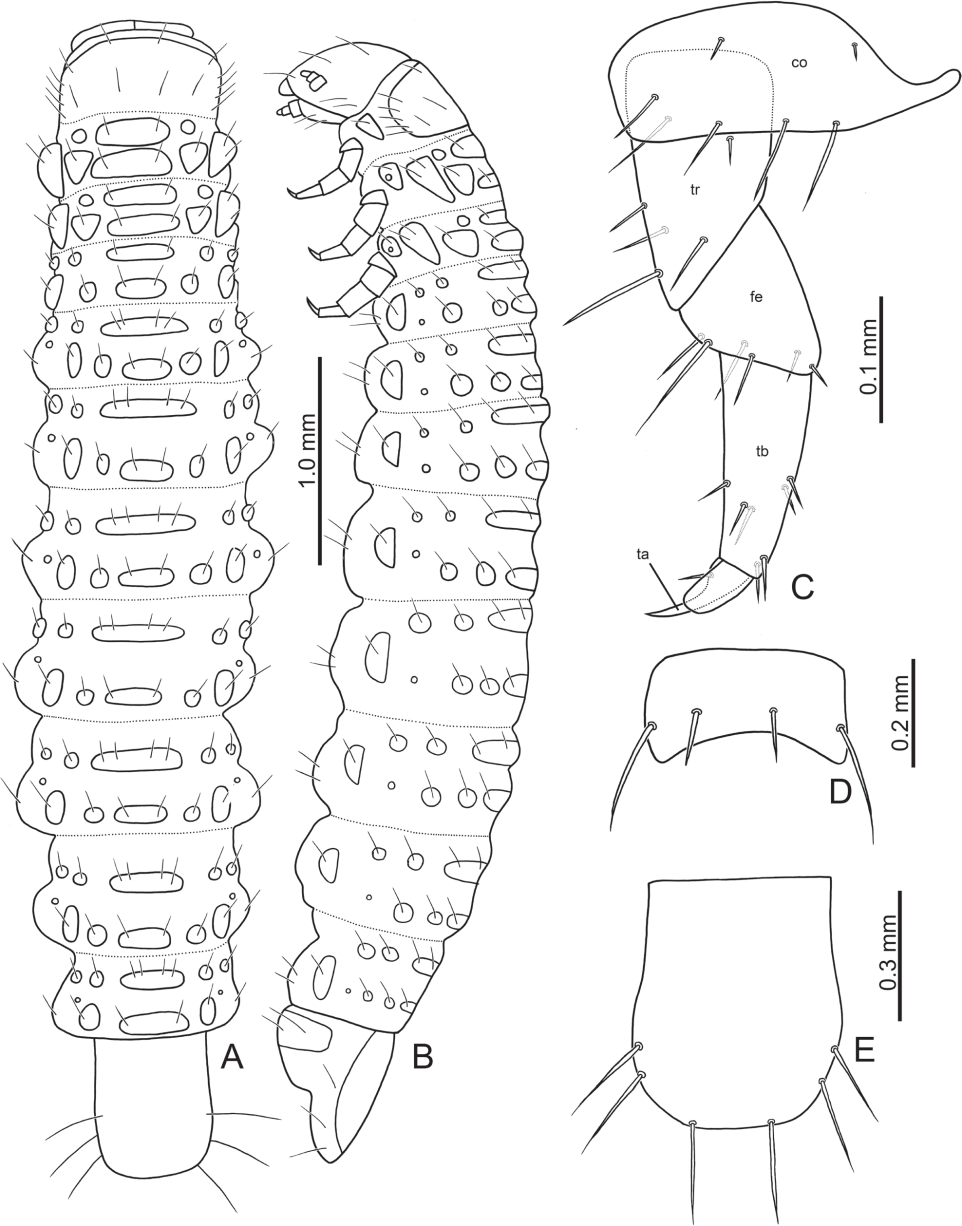
*Nisotragemella* (Erichson), third-instar larva **A** larva in dorsal view **B** larva in lateral view **C** leg **D** abdominal ventrite IX **E** pygopod. Abbreviations: co- coxa; fe- femur; ta- tarsungulus; tb- tibia; tr- trochanter.

**Figure 9.**
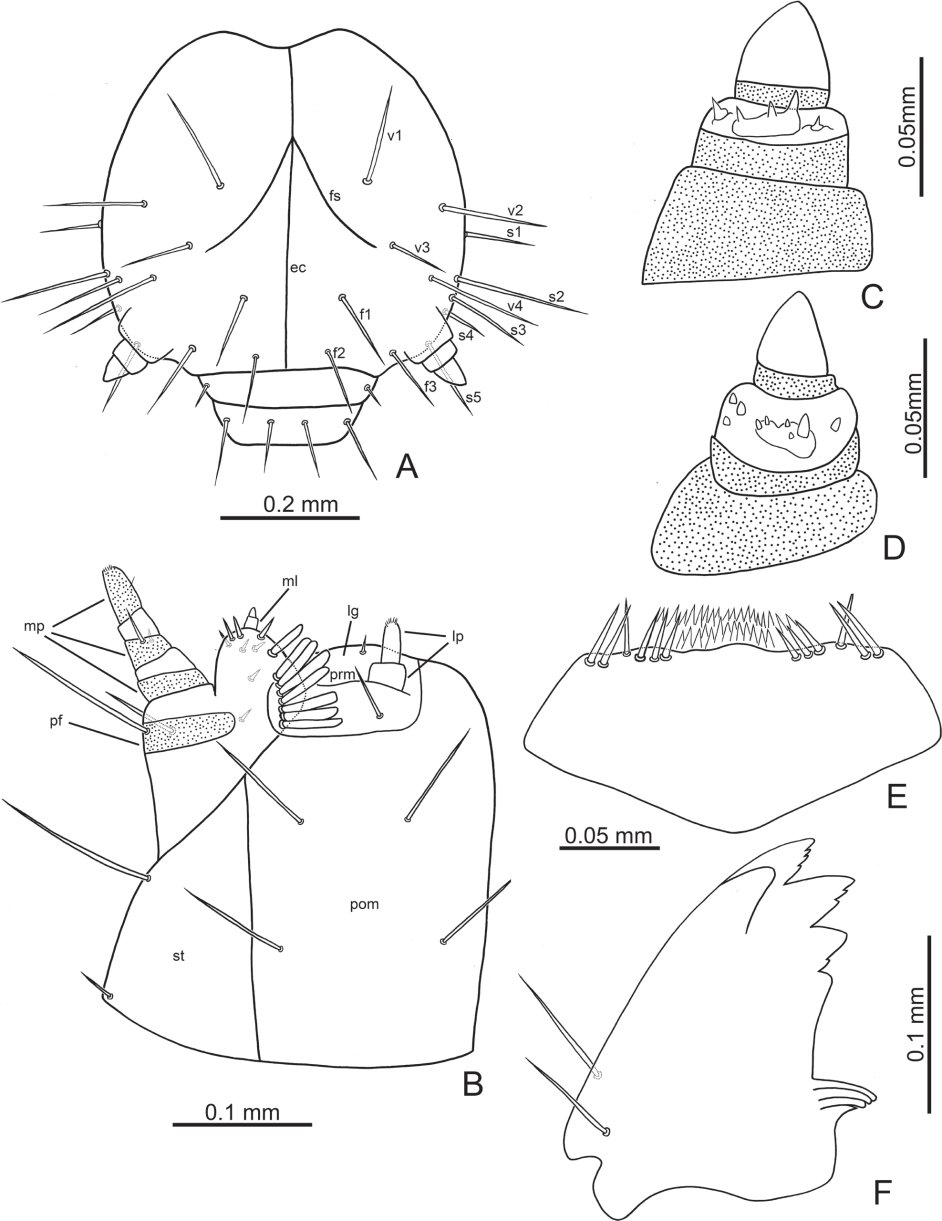
*Nisotragemella* (Erichson), third-instar larva **A** head **B** maxilla and labium **C** antenna, in lateral view **D** antenna, in dorso-lateral view **E** epipharynx **F** mandible. Abbreviations: ec- endocarina; f1–f3- setae on frontal area; fs- frontal suture; lg- ligula; lp- labial palp; ml- mala; mp- maxillary palp; pf- palpifer; pom- postmentum; prm- prementum; s1–s5- setae on sides of head; st- stripes; v1–v4- setae on vertex.

Third-instar larvae. Length 4.8 mm, width 1.0 mm, cylindrical, cream colored, with tubercles well-developed, head dark brown (Fig. 8A, B). Head (Fig. 9A) hypognathous, rounded, strongly sclerotized; surface generally smooth. Endocarina (ec) visible, extending into clypeus. Frontal suture (fs) V-shaped, starting from apical 1/3 of endocarina; apically abbreviated halfway between endocarina and lateral margin. Vertex with four pairs of setae (v1–v4). Frons with three pairs of setae (f1–f3). Sides with five pairs of setae (s1–s5). Clypeus transverse, apical margin straight, with one pair of small setae at sides. Labrum transverse, apical margin irregular, with two pairs of long setae; epipharynx (Fig. 9E) with several setae at sides, and flattened, apically pointed setae. Mandible (Fig. 9F) robust; five-toothed, with tiny serrations on mesal margins of teeth; two setae on opposite sides; mola reduced; prostheca composed of several flattened, slender setae. Maxillary palpus (mp) (Fig. 9B) three-segmented, palpomere II with one long seta at one side and one short seta at opposite side; palpifer (pf) with one long seta at opposite sides; mala (ml) with two-segmented conical sensorium and surrounding by three pairs of short setae, mesal side margined with one row of elongate, flattened setae with rounded apices; stipes (st) with one long seta and one short seta. Labial palp (lp) two-segmented, palpomere II cylindrical, palpomere I much wider than II; ligula (lg) rounded, with one pair of short setae; prementum (prm) with one pair of long setae; postmentum (pom) with two pair of long setae. Stemmata reduced and invisible. Antenna (Fig. 9C, D) three-segmented, one tubercle with one prominent and several tiny conical sensoria on antennomere II, one large conical sensorium on antennomere III.

**Figure 10.**
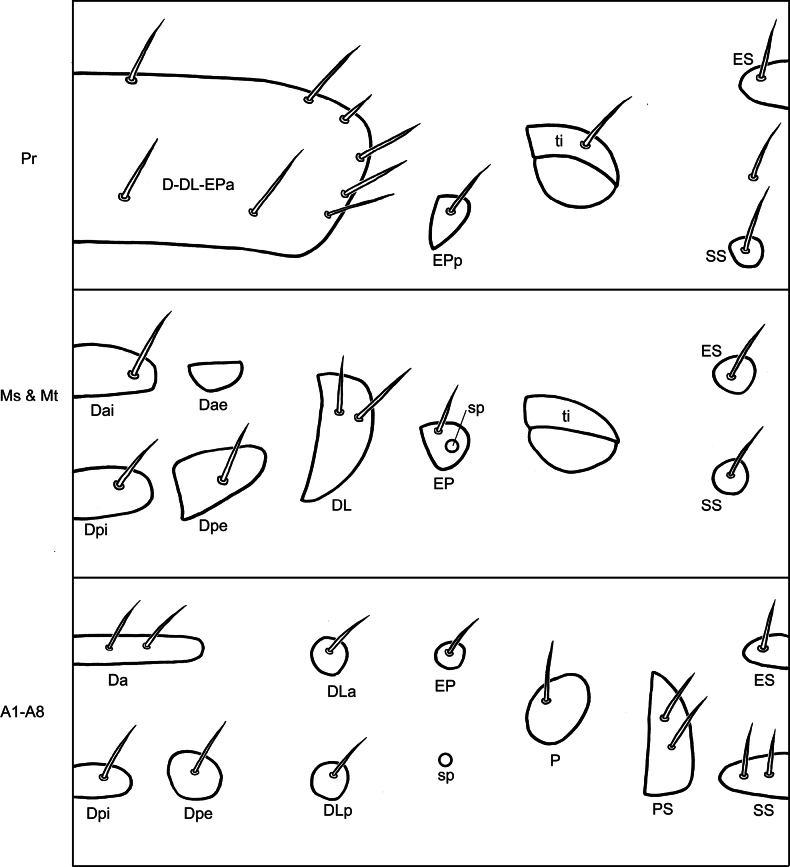
*Nisotragemella* (Erichson), third-instar larva: location of tubercles and body chaetotaxy. Abbreviations: Pr- prothorax; Ms- mesothorax; Mt- metathorax; A1–A8- abdominal segments I–VIII.

Prothorax (Fig. 10) with dorsal, dorso-lateral, and anterior epipleural tubercles fused, D-DL-EPa type; basal and apical margins with two pairs of setae, lateral margins with four pairs of setae; epipleural region with one small tubercle EPp, with one seta; trochantin (ti) with one seta; sternal regions with two tubercles: ES and SS, ES not divided medially, with one pair of setae, SS separated, with one seta on each tubercle; one additional seta between ES and SS. Meso- and metathoraces with dorsal tubercle divided into Dai, Dae, Dpi, and Dpe; with one pair of setae on each tubercle, except Dae; DL tubercle not divided, with two setae; epipleuron with one tubercle, EP, surrounding spiracle, with one seta; trochantin (ti) without setae; sterna with two tubercles, ES and SS, separated medially, with one seta on each tubercle. Legs (Fig. 8C) five-segmented, coxa (co) with four long setae apically and two short setae basally; trochanter (tr) with four long mesal setae, one long seta apically, and one short seta at lateral area; femur (fe) with three pairs of apical setae on opposite; tibia (tb) with one short mesal seta near margin, two pairs of setae near outer margin, and one pair of setae apically; tarsungulus (ta) slightly curved, with broad base, with one mesal seta near margin of broad base.

Abdominal segment I–VIII (Fig. 10) with dorsal tubercles divided into three tubercles: Da, Dpi, and Dpe, Da and Dpi not divided medially, Da with two pairs of setae, Dpi with one pair of setae, Dpe with one seta; dorso-lateral region with two tubercles: DLa and DLp, with one seta on each tubercle; epipleural tubercle with one seta separated from spiracle, spiracle rounded, tubercle P with one seta; sternal region with three tubercles: PS, ES, and SS; ES and SS not divided medially, ES with one pair of setae but SS with two pairs of setae; PS large and with two setae. Abdominal segment IX covered by pygopod dorsally (Fig. 7E); pygopod oblong, slightly and apically widened, with three pairs of setae margined apically; ventral region (Fig. 8D) sclerotized and not divided, with two pairs of setae.

**Figure 11.**
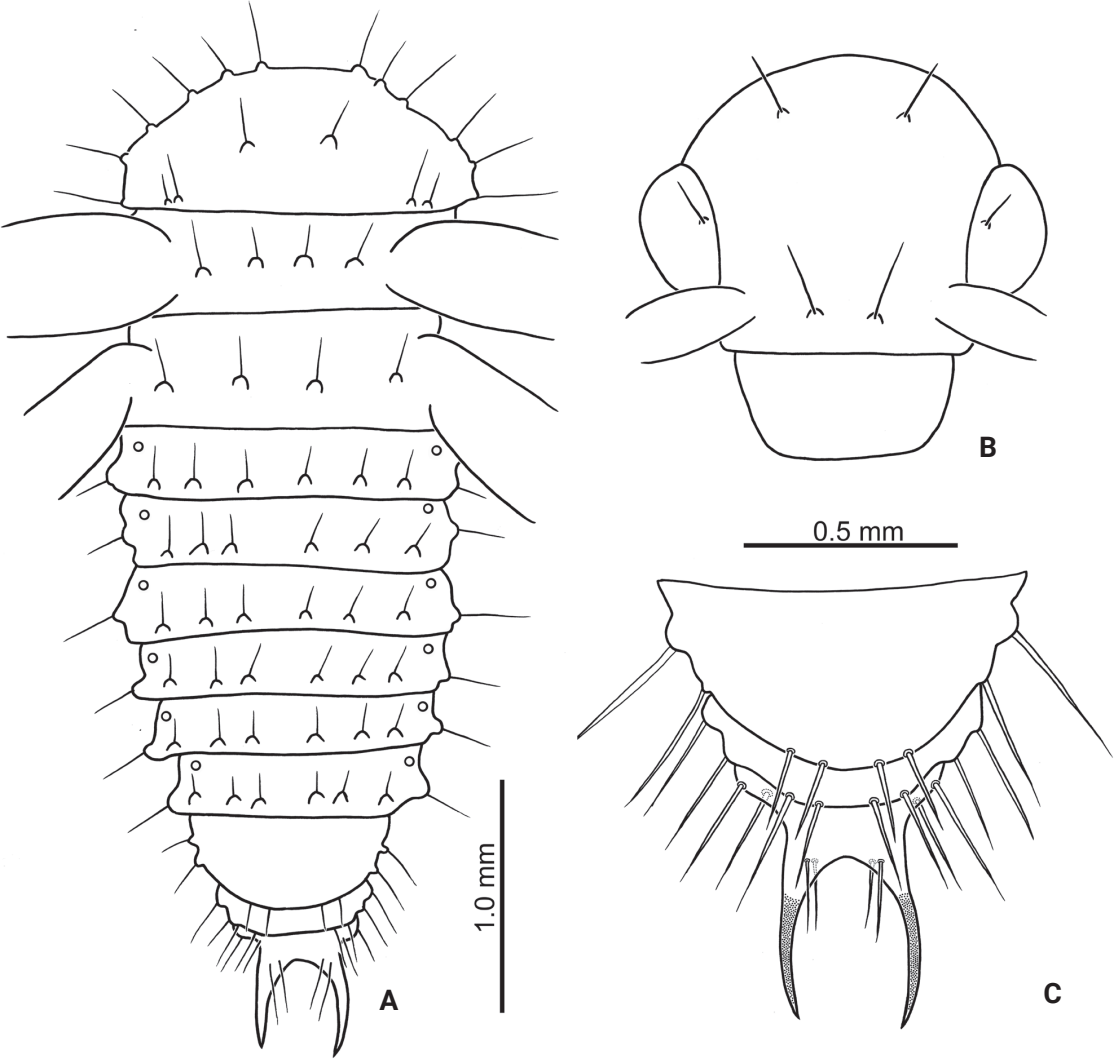
*Nisotragemella* (Erichson), pupa **A** dorsal view **B** head **C** abdominal segments VII–IX.

**Pupa.** Length 3.8 mm; width 1.4 mm, yellowish white. Head (Fig. 11B) with three pairs of setae: one pair on vertex, one pair on mesal margins of eyes, the other pair between antennae. Prothorax (Fig. 11A) with five pairs of setae on outer margin; one pair on baso-lateral angles; two pairs near basal margin and close to each other, outer pair shorter; one pair at central part near middle line. Meso- and metathoraces with two pairs of setae. Abdominal segment I–VIII (Fig. 11A) with four pairs of setae; setae on abdominal segment I–VI near posterior margin; VII (Fig. 11C) expanded posteriorly, setae along posterior margin; VIII similar to VII but narrower; IX with apical processes sclerotized at apex and strongly pointed, two pairs of setae at sides, two pairs on base of apical processes, one on each, dorsal and ventral surfaces, respectively. Bases of femora of front and middle legs with three pairs of setae, but only two pairs of setae on bases of femora of hind legs.

#### Host plants.

Malvaceae: *Hibiscusrosa-sinensis* (Bryant 1941 and current study) (Fig. 11F), *H.tiliaceus* (current study), *Urenalobata* (Kimoto 2003), and Lamiaceae: *Mesonachinensis* (current study). Takizawa (1979) recorded two species of Urticaceae (Boehmerianiveavar.nivea and *Gonostegiahirta*) as host plants for Taiwanese populations of *Nisotraorbiculata* (= *N.gemella*) but this may be incorrect because flea beetles collected from both plants belong to the genus *Euphitrea* Baly. Both plants were also regarded as host plants for *Neorthaea* (= *Euphitrea*) *nisotroides* Chen and *Neorthaeaflavicornis* Chen in the same paper (Takizawa 1979). We assessed five plants for adults of *Nisotragemella*, which fed originally on *Mesonachinensis* in the laboratory. These five were *Hibiscusrosa-sinensis*, *H.tiliaceus*, *Urenalobata* (Malvaceae), Boehmerianiveavar.nivea (Urticaceae), and *Menthacanadensis* (Lamiaceae). They did not feed on leaves of Boehmerianiveavar.nivea or *Menthacanadensis*.

#### Biology.

**Figure 12.**
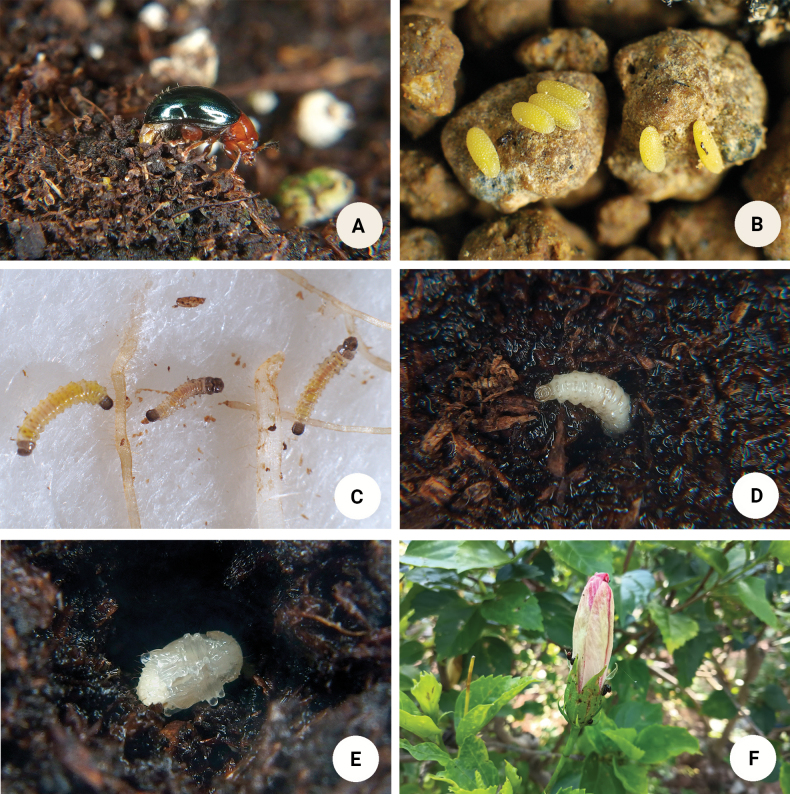
*Nisotragemella* (Erichson) **A** female depositing eggs on soil **B** eggs **C** larvae feeding on roots **D** mature larvae crawling into soil and constructing underground chamber for pupation **E** pupa **F** adults attacking *Hibiscusrosa-sinensis*.

Females deposited eggs on the soil or leaf litter (Fig. 12A). Each egg (Fig. 12B) was oblong and pale yellow, 1.0 mm in length and 0.5 mm in width. Larvae hatched after 10–14 days, and have three instars Larvae were pale yellow with black heads, pronota, and pygopods (Fig. 12C). They fed on roots. Larval durations varied from 25–46 days. Mature larvae (Fig. 12D) crawled into the soil and constructed underground chambers for pupation. Pupal stage (Fig. 12E) durations were 7–8 days. Adults fed by cutting the leaf lamina. They jumped promptly when disturbed. Adults remained on the adaxial side of leaves.

#### Remarks.

The specimens collected from Palawan, Philippines (Medvedev 1993) and Vietnam, were misidentified and represent *Nisotrachrysomeloides*. The same error has been made by other taxonomists.

#### Distribution.

Cambodia, China, India (including Andaman and Nicobar islands), Indonesia (Sumatra, Java, Sulawesi), Laos, Malaysia, Myanmar, Nepal, Papua New Guinea, Philippines (Luzon), Singapore, Thailand, Taiwan, and Vietnam.

### ﻿ Nisotra nigripes

Taxon classificationAnimaliaColeopteraChrysomelidae

Jacoby

C75CAED3-ED91-5BE5-B415-13D9F8CBFA3F

**Figure 13.**
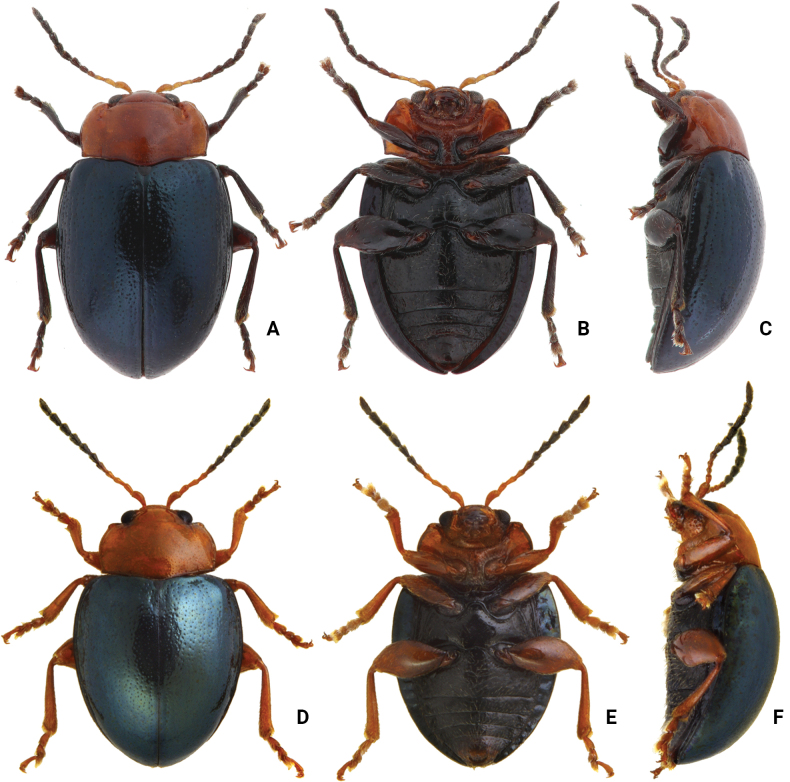
Habitus of *Nisotranigripes* Jacoby **A** female, from Myanmar, dorsal view **B** ditto, ventral view **C** ditto, lateral view **D** male, from Taiwan, dorsal view **E** ditto, ventral view **F** ditto, lateral view.

**Figure 14.**
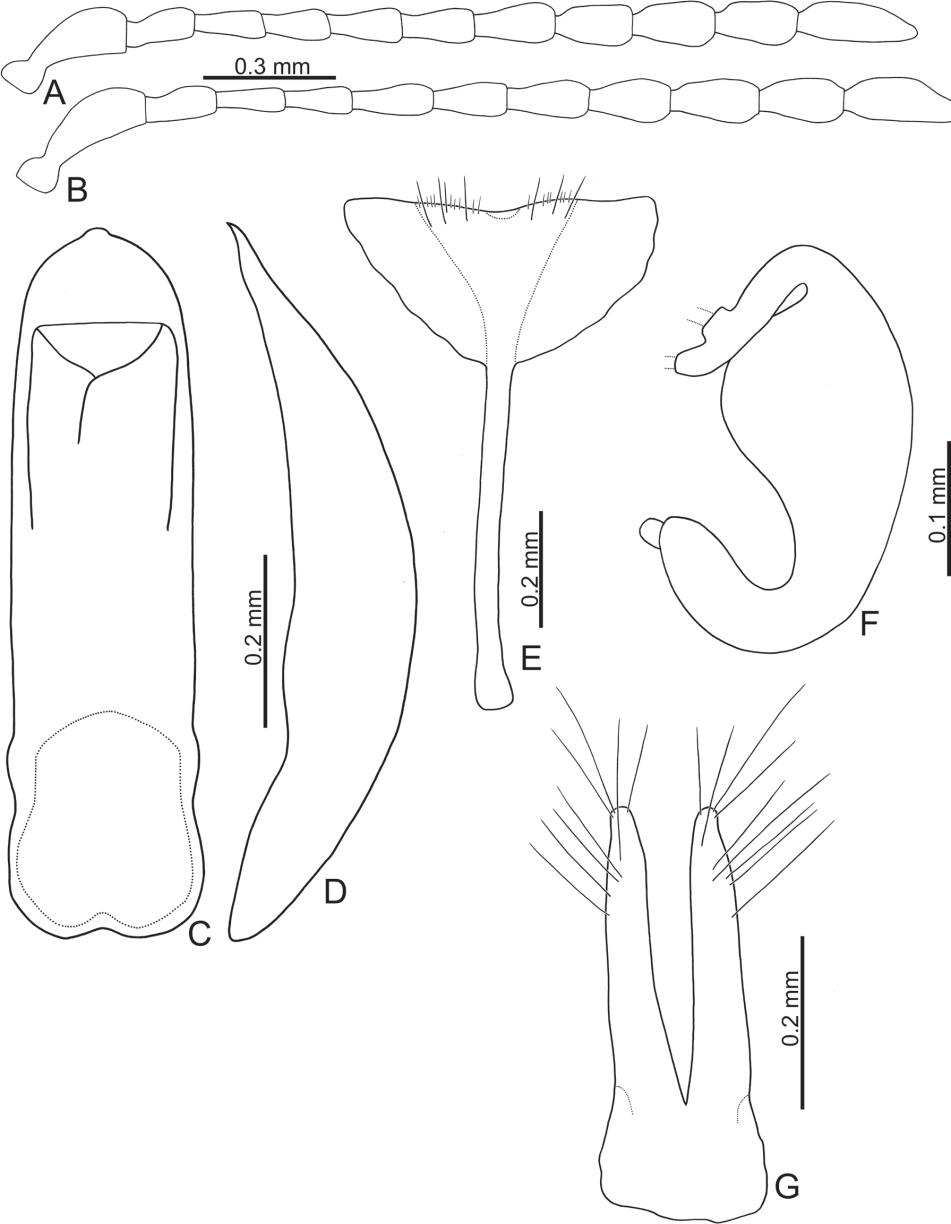
*Nisotranigripes* Jacoby, adult **A** antenna, male **B** antenna, female **C** aedeagus, dorsal view **D** aedeagus, lateral view **E** abdominal ventrite VIII, female **F** spermatheca **G** gonocoxae.

Figs 6O, P , 13 , 14

Nisotra nigripes Jacoby, 1894: 293 (Myanmar); Medvedev 2000: 21 (Nepal); Zhang and Yang 2007: 844 (China: Yunnan).
Nisotra orbiculata sensu Kimoto 1970: 215 (Taitung: Chipen (知本); Chiayi: Fenchihu (奮起湖)); Kimoto 1989: 263 (Kaohsiung: Liu Kui (六龜)).

#### Type.

***Holotype*** (sex undetermined, based on photographs, MCZC, fixed by monotypy) (Fig. 2O, P): “Ruby Mines / U. B. [p, w] // Type [p] / 18565 [h, r] // Nisotra / nigripes / Jac. [h, b]”.

#### Additional material examined.

**Laos.** Vientiane: 1♂ (NHMUK), 1 km W Vang Vieng, 15.VIII.2004, leg. M. Geiser; **Myanmar.** 3♀ (NHMUK), Toungoo, coll. Andrewes, 1922-221; 1♂ (NHMUK), Ruby Mines, leg. Doherty, Fry Coll., 1905.100; Kachin State: 2♂, 2♀ (NHMUK), Nam Tamai, 2.VIII.1938, leg. R. Kaulback; Naga: 1♂, 1♀ (SEHU), Somura, 1–2.V.2005, leg. A. Abe; **Taiwan.** Chiayi: 1♂ (KMNH), Fenchihu (奮起湖), 12.IV.1965, leg. T. Saigusa; Hsinchu: 6♂, 2♀ (TARI), Tahunshan (大混山), 24.II.2009, leg. S.-F. Yu; 1♂ (TARI), Talu trail (大鹿林道), 22.X.2008, leg. H.-J. Chen; Kaohsiung: 1♀ (TARI), Chungchihkuan (中之關), 16.IV.2012, leg. L.-P. Hsu; 2♀ (KMNH), Liu Kui (六龜), 31.III.1986, leg. K. Baba; 2♂, 3♀ (TARI), Namahsia (納瑪夏), 1.IX.2012, leg. Y.-T. Chung; 1♀ (TARI), Peitawushan (北大武山), 27.V.2013, leg. Y.-T. Chung; 2♀ (TARI), same but with “1.IX.2016”; 1♂ (TARI), Shihshan logging trail (石山林道), 1–3.X.2008, leg. M.-H. Tsao; 2♂, 3♀ (TARI), Tengchih (藤枝), 2–5.VI.2008, leg. C.-F. Lee; 1♂ (TARI), same locality, 8.VI.2013, leg. W.-C. Liao; Nantou: 2♂, 1♀ (NMNS), Hsitou (溪頭), 21.VIII.2006, leg. W. T. Jin; 1♂ (KMNH), Lushan Wenchuan (廬山溫泉), 6.VI.1976, leg. H. Makihara; 1♂ (TARI), same locality, 27–31.V.1980, leg. K. S. Lin & L. Y. Chou; 3♂, 2♀ (TARI), Tungpu (東埔), 28.IV. –2.V.1981, leg. T. Lin & C. J. Lee; 1♀ (TARI), same locality, 18–23.XI.1981, leg. T. Lin & W. S. Tang; 1♂, 3♀ (TARI), same locality, 19–23.VII.1982, leg. L. Y. Chou & T. Lin; 2♂, 2♀ (TARI), same locality, 16–20.IV.1984, leg. K. C. Chou & C. H. Yung; 1♂, 6♀ (TARI), same locality, 23–27.VII.1984, leg. K. C. Chou & C. H. Yang; Pingtung: 6♂, 4♀ (TARI), Laii (來義), 23.IV.2008, leg. W.-T. Liu; 2♂, 4♀ (TARI), Wutai (霧台), 12.IV.2009, leg. U. Ong; Taichung: 1♂ (TARI), Chiapotai (佳保台), 14–18.X.1980, leg. K. S. Lin & C. H. Wang; Taitung: 2♂, 1♀ (KMNH), Chipen (知本), 10.VIII.1966, leg. H. Kamiya; 1♂, 2♀ (TARI), Lichialintao (利嘉林道), 24.IV.2008, leg. C.-L. Hsiao; 2♂, 1♀ (TARI), Liyuan (栗園), 28.III.2014, leg. W.-C. Huang; **Thailand.** Siam: 2♀ (KMNH), Tak, 20.VIII.1961.

#### Redescription.

**Adults.** Length 3.6–4.4 mm, width 2.3–2.7 mm (*n* = 92). General color yellowish brown (Fig. 13A–C); elytra, meso- and metathoracic and abdominal ventrites metallic purple; legs black. four basal antennomeres I–IV yellowish brown, V dark brown, VI–XI black. Antennae (Fig. 14A) filiform in males, ratios of lengths of antennomeres I to XI 1.0: 0.5: 0.5: 0.5: 0.6: 0.5: 0.6: 0.6: 0.6: 0.6: 0.9; ratios of length to width from antennomeres I to XI 2.9: 2.1: 2.5: 2.1: 2.2: 2.1: 2.0: 1.9: 1.8: 1.9: 3.1; similar in females, ratios of lengths of antennomeres I to XI (Fig. 14B) 1.0: 0.4: 0.4: 0.4: 0.5: 0.5: 0.5: 0.5: 0.6: 0.6: 0.8; ratios of length to width from antennomeres I to XI 3.3: 2.2: 2.8: 2.4: 2.7: 2.1: 2.2: 1.9: 2.1: 1.9: 2.8. Pronotum 1.8–1.9 × wider than long; disc shining, with sparse, fine punctures, less convex; longitudinal groove on each side of apical margin shallow, with several coarse punctures along longitudinal groove; short and shallow longitudinal groove on basal margin; lateral margins rounded; apical margins slightly concave; basal margin medially convex. Elytra 1.2 × longer than wide; disc with coarse punctures arranged into longitudinal lines, with fine punctures between coarse punctures; lateral margins rounded, narrowed behind middle. Aedeagus (Fig. 14C, D) wide, ~ 4.2 × longer than wide; parallel sided, subapically narrow, apex with one median rounded process; slightly curved in lateral view, apex directed inward; tectum membranous, internal sac without stout setae. Endophallic spiculae reduced. Gonocoxae (Fig. 14G) longer than wide, and basally connected; each gonocoxa subapically narrowed and apex truncate, and curved outwards, with eight or nine long setae along apical and outer margins. Ventrite VIII (Fig. 14E) with apex weakly sclerotized, with one semicircular membranous area at middle of apical margin and several long setae in a transverse line near apical margin, with several setae along apical margin, both types of setae absent medially, spiculum extremely long. Spermathecal receptaculum (Fig. 14F) strongly swollen; pump long and curved, with small apical process; spermathecal duct sclerotized, short after base of spermathecal gland.

#### Variation.

Adults from Taiwan have yellowish brown legs (Fig. 13D–F) that are different from those of the Asian continent, which possess black legs.

#### Diagnosis.

Most adults of *N.nigripes* Jacoby are similar to those of *N.chrysomeloides* but differ in possessing black legs (yellowish brown legs in others). However, Taiwanese populations of *N.nigripes* is not distinguishable from those of *N.chrysomeloides*, which is not recorded from Taiwan. In males of *N.nigripes*, the widely rounded apex of the aedeagus bearing a small process at the middle (Fig. 14C) differs from the truncate apex and small process in *N.gemella* (Fig. 7C) and *N.dohertyi* (Fig. 5C), and acute apex in *N.chrysomeloides* (Fig. 4C). The slightly curved aedeagus in lateral view (Fig. 14D) differs from the moderately curved aedeagus in *N.gemella* (Fig. 7D) and *N.chrysomeloides* (Fig. 4E), and strongly curved aedeagus in *N.dohertyi* (Fig. 5E). The membranous tectum (Fig. 14C) differs from the sclerotized tectum in *N.chrysomeloides* (Fig. 4C). In females of *N.nigripes*, laterally directed apices of and gonocoxae (Fig. 14G) are different from the straight gonocoxae in *N.gemella* (Fig. 7G), dorsally directed apices in *N.chrysomeloides* (Fig. 4H), and inwardly directed apices in *N.dohertyi* (Fig. 5I). The setae of abdominal ventrite VIII, with one transverse line of long setae inside the apical margin and dense short setae along the apical margin (Fig. 14E) differs from the presence of several pairs of long setae along the apical margin in *N.dohertyi* (Fig. 5F, G).

#### Host plant.

Adults in Taiwan feed on leaves of *Hibiscustaiwanensis*, which is an endemic plant.

#### Distribution.

China, Laos, Myanmar, Taiwan, and Thailand.

Zhang and Yang (2007) provided a key to Chinese species of *Nisotra*. We think most of the key is appropriate, but lengths of bodies and some coloration characters are too variable for reliable diagnoses. It is modified to include species from Taiwan as follows:

**Table:** 

1	Distinct longitudinal groove on each side of anterior margin of pronotum; pronotum less convex	**2**
–	Inconspicuous longitudinal groove on each side of anterior margin of pronotum	**5**
2	Body ovate (1.1 × longer than wide); confused punctures on elytra; pronotum dull and with micro-reticulation	***N.dohertyi* (Maulik)**
–	Body oblong (1.2 × longer than wide); punctures on elytra arranged into paired longitudinal rows; pronotum shining and lacking micro-reticulation	**3**
3	Legs black	***N.nigripes* Jacoby**
–	Legs yellowish brown	**4**
4	Specimens collected from Taiwan	***N.nigripes* Jacoby**
–	Specimens collected from other areas	***N.chrysomeloides* Jacoby**
5	Longitudinal groove of each side of basal margin of pronotum inconspicuous and short, < 1/5 of pronotum	***N.gemella* (Erichson)**
–	Longitudinal groove of each side of basal margin of pronotum distinct and long, > 1/5 of pronotum	***N.xinjiangana* Zhang & Yang**

## ﻿Discussion

*Nisotrachrysomeloides*, *N.dohertyi*, *N.gemella*, and *N.nigripes* represent more than 95% of museum specimens in historical collections collected from Southeast Asia, China, and Taiwan. Many identified specimens are misidentified, probably because few diagnostic characters can be used for reliable species identifications. Moreover, diagnostic characters can be variable. Doubtfully identified specimens must be dissected for identification. Distributions of each will require updating based on the present study. *Nisotragemella* recorded from Taiwan is confirmed, and *N.nigripes* is newly recorded from Taiwan. Only aedeagi and spermathecae were used as diagnostic characters in the former studies (e.g., Scherer 1969; Zhang and Yang 2007). The results show that spermathecae are less diagnostic in species identities but more diagnostic in supraspecific classification. Abdominal ventrite VIII in females and gonocoxae are diagnostic for species identities. We suggest that both structures are needed in current and future taxonomy.

*Nisotragemella* is one of the most widespread flea beetles of the genus ocurring from China and Taiwan to Papua New Guinea. Such widespread distribution might be result of two host plants. *Hibiscustiliaceus* are adapted to the sea-shore habits. The colonization of *N.gemella* on many islands and coastal areas across the Pacific and Indian Oceans is likely associated with this peculiar feature of their host. In Taiwan, adults are not only found in mainland, but also Kinmen Island (金門島) and Beigan Island (北竿島). Its distribution is similar to another leaf beetle, *Pholaoctodecimguttata* (Fabricius, 1775) (Lee and Geiser 2023) adapted to seashore habitats. The second plant is *H.rosa-sinensis* which is a popular ornamental plant in Asia. Humans planted substantial populations surrounding structures and gardens. Thus, adults have become common due to associations with the plant. In the present study we found this species also fed on one important crop, *Mesonachinensis*. It has become a major pest where mesona has been planted in large areas. Clarification of species identity will contribute to managing this pest in the future.

*Nisotranigripes* is not a common species and was not studied by Scherer (1969) due to lack of specimens. Zhang and Yang (2007) were the first to illustrate the male aedeagus based on specimens collected from Yunnan. In Taiwan, adults of *N.nigripes* are more common at many localities than those of *N.gemella*. This is a result of the common occurrence of its host plant *Hibiscustaiwanensis* in mountainous areas. By contrast, although the host plants for *N.gemella* are common in lowlands of Taiwan and around human habitations, adults of *N.gemella* are not common, probably due to sensitivity to human disturbance or chemicals. A host plant shift to *Mesonachinensis* seems to have ensured survival of *N.gemella* populations.

## Supplementary Material

XML Treatment for Nisotra chrysomeloides

XML Treatment for Nisotra dohertyi

XML Treatment for Nisotra gemella

XML Treatment for Nisotra nigripes

## ﻿Additional information

### Author ORCIDs

Chi-Feng Lee https://orcid.org/0000-0003-1996-0557

Ming-Yao Chiang https://orcid.org/0000-0002-1514-7086

### Data availability

All of the data that support the findings of this study are available in the main text.
